# Flexibility Enhances Reactivity: Redox Isomerism and Jahn–Teller Effects in a Bioinspired Mn_4_O_4_ Cubane Water Oxidation Catalyst

**DOI:** 10.1021/acscatal.1c03566

**Published:** 2021-10-18

**Authors:** Ludwig Schwiedrzik, Vera Brieskorn, Leticia González

**Affiliations:** Institute of Theoretical Chemistry, Faculty of Chemistry, University of Vienna, Währinger Straße 17, 1090 Vienna, Austria

**Keywords:** artificial photosynthesis, polyoxometalate, Jahn−Teller axis, O−O bond formation, density functional theory

## Abstract

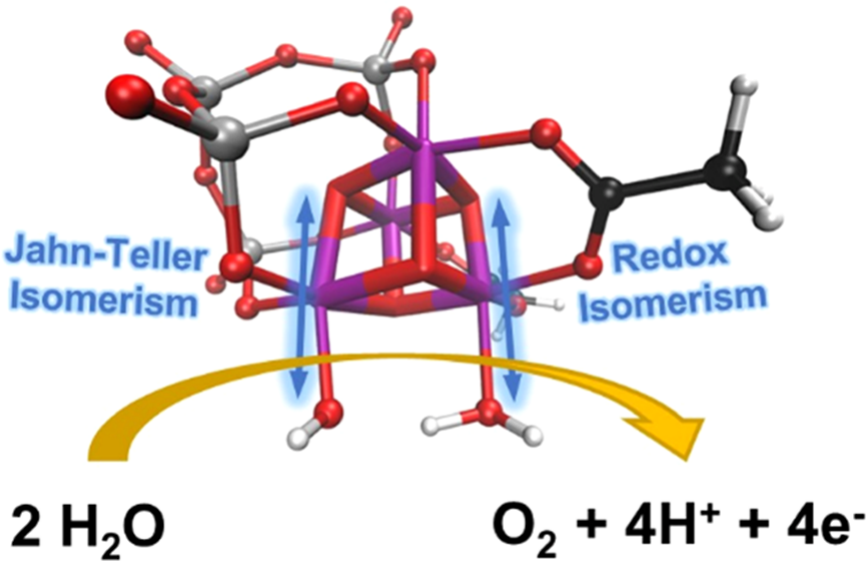

Understanding how water oxidation to molecular oxygen proceeds in molecular metal-oxo catalysts is a challenging endeavor due to their structural complexity. In this report, we unravel the water oxidation mechanism of the highly active water oxidation catalyst [Mn_4_V_4_O_17_(OAc)_3_]^3–^, a polyoxometalate catalyst with a [Mn_4_O_4_]^6+^ cubane core reminiscent of the natural oxygen-evolving complex. Starting from the activated species [Mn_4_^4+^V_4_O_17_(OAc)_2_(H_2_O)(OH)]^1–^, we scrutinized multiple pathways to find that water oxidation proceeds via a sequential proton-coupled electron transfer (PCET), O–O bond formation, another PCET, an intramolecular electron transfer, and another PCET resulting in O_2_ evolution, with a predicted thermodynamic overpotential of 0.71 V. An in-depth investigation of the O–O bond formation process revealed an essential interplay between redox isomerism and Jahn–Teller effects, responsible for enhancing reactivity in the catalytic cycle. This is achieved by redistributing electrons between metal centers and weakening relevant bonds through Jahn–Teller distortions, introducing flexibility to the otherwise rigid cubane core of the catalyst. These mechanistic insights are expected to advance the design of efficient bioinspired Mn cubane water-splitting catalysts.

## Introduction

Climate change caused by the emission of anthropogenic CO_2_ and other greenhouse gases into the atmosphere is one of the greatest challenges facing humanity today.^1,2^ Among the many technologies being developed to reduce CO_2_ emissions, artificial water splitting promises to replace fossil fuels with a clean-burning alternative, hydrogen gas.^3,4^ Inspired by the natural process of photosynthesis, artificial water splitting aims to produce oxygen and hydrogen according to
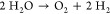
1consisting of the half-reactions
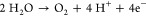
2
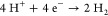
3wherein eq 2 is referred to as water oxidation and eq 3 as hydrogen evolution.^5,6^ Of the two, water oxidation is thermodynamically more challenging, as it comprises four one-electron oxidations and four deprotonations, usually assumed to be coupled.^6^ This has inspired a massive research effort to come up with ever-more effective water oxidation catalysts (WOCs).^6−13^

In the development of synthetic molecular WOCs, a number of central design criteria have emerged:^6,10^ (i) the WOC should catalyze water oxidation at a low thermodynamic overpotential, that is, the overall reaction potential should be overcome in four equal steps;^6,14,15^ (ii) the WOC should be stable under the oxidative conditions typically found in experimental photo- or electrocatalytic water-splitting setups;^6,16^ (iii) earth-abundant elements should be used for the metal centers to minimize the cost and environmental impact of future industrial-scale usage;^16^ and (iv) every synthetic WOC is judged by its activity, with the ultimate goal of approaching or even surpassing the natural oxygen-evolving complex (OEC).^17,18^

**Scheme 1:**
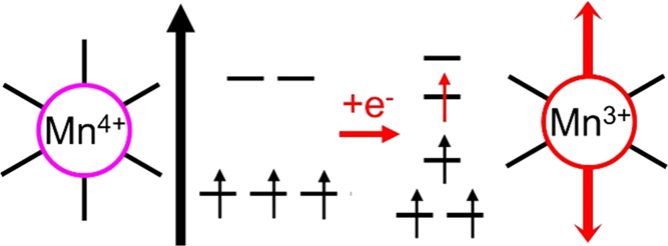
Reduction of Mn^4+^ Leads to the Emergence of Jahn–Teller (JT) Distortions Due to Splitting of the Octahedral Ligand Field in Mn^3+^, Resulting in the Elongation and Weakening of One Bond Axis (Marked in Red)

Given this variety of difficult-to-reconcile goals, it is not surprising that the design of many WOCs is inspired by nature, attempting to copy one or several aspects of the OEC. As the OEC is centered on a Mn_3_CaO_4_ cubane structure with a dangling fourth Mn center,^19^ such cubane structures have received considerable attention. In particular, Co cubane WOCs have been extensively studied both experimentally and theoretically.^13,17,20^ It was found that terminal Co-oxo or -oxyl groups play a central role in O–O bond formation on Co cubanes, with a variety of water oxidation cycles being described.^21−26^ The relatively high water oxidation activity of these systems has been linked to the ability of cubanes to flexibly redistribute electrons between the metal centers.^23−25^ A number of Mn cubane WOCs have also been investigated, often as model systems for the OEC.^18,27−31^ In these model systems, it has been noted that Jahn–Teller (JT) effects present on Mn^3+^ centers can lead to significant distortions of the cubane^32−40^ and even influence ligand exchange kinetics or oxidation pathways.^41,42^ Such JT distortions arise from d^4^ ions such as Mn^3+^ due to the energetically favorable splitting of the octahedral ligand field, resulting in the elongation and concomitant weakening of one bond axis (see Scheme 1).

**Figure 1:**
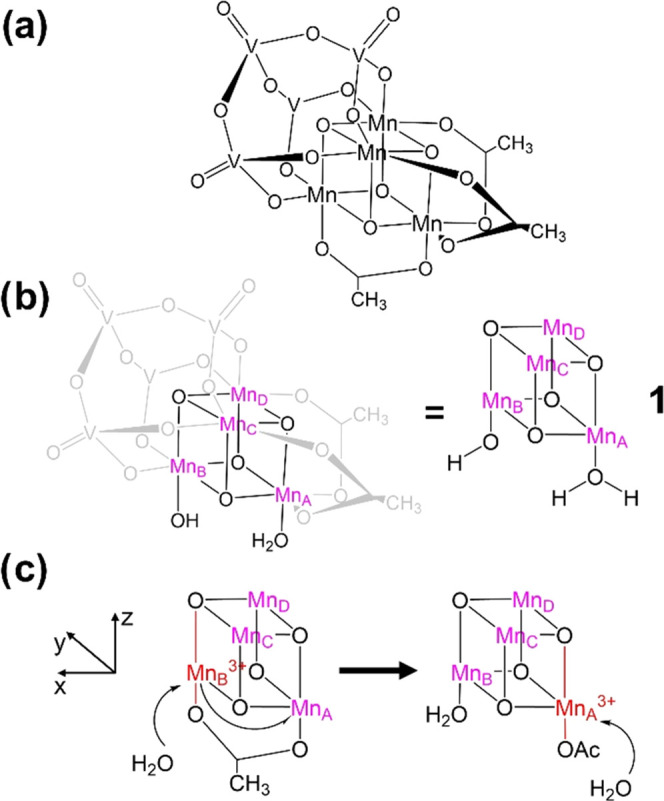
(a) Structural formula of the pristine water oxidation catalyst [Mn_4_V_4_O_17_(OAc)_3_]^3−^. (b) Activated species [Mn_4_^4+^V_4_O_17_(OAc)_2_(H_2_O)(OH)]^1–^ (**1**), with Mn^4+^ centers labeled A–D in purple (left). Abbreviated structure of **1** showing only the cubane core and reactive ligands (right). (c) Intermediates during catalyst activation with Mn^3+^ centers and Jahn–Teller (JT) axes marked in red: a redox isomerization (half arrow, left) lowers the barrier for the second water attack (full arrow, right), as the Mn_A_^3+^-OAc bond is weakened by JT distortion.

Here, we focus on a promising bioinspired catalyst, [Mn_4_V_4_O_17_(OAc)_3_]^3–^, a highly active synthetic WOC with a TON > 12 000 and a TOF > 200 min^–1^.^43,44^ It consists of a Mn_4_O_4_ cubane core, surrounded on three sides by a multidentate V_4_O_13_ vanadate ligand and three acetate ligands on the remaining sides (Figure 1a). A recent theoretical and experimental study^45^ uncovered the activation mechanism of the precatalytic species [Mn_2_^3+^Mn_2_^4+^V_4_O_17_(OAc)_3_]^3–^, consisting of a one-electron oxidation, ligand exchange accompanied by deprotonation, and further one-electron oxidation, resulting in the activated species [Mn_4_^4+^V_4_O_17_(OAc)_2_(H_2_O)(OH)]^1–^ (**1**) (Figure 1b). In that work, **1** was assigned an experimental redox potential of 1.25 V, which is below the onset of water oxidation observed at 1.6 eV under electrochemical conditions, suggesting that **1** initiates the water oxidation cycle. It was also found that during the acetate-to-water ligand exchange, the first water molecule attacks along the JT-distorted bond axis of Mn_B_^3+^ (see Figure 1c), taking advantage of the weaker Mn^3+^–OAc bond present at that metal center. A subsequent electron transfer from Mn_B_ to Mn_A_ allows the second water molecule to also attack a weak JT-distorted bond, resulting in a much lower reaction barrier than an attack at a Mn^4+^ center would (8.0 vs 26.7 kcal/mol). These findings hinted at a possible role of the JT axes controlling the reactivity of [Mn_4_V_4_O_17_(OAc)_3_]^3–^.^45^ It is well known that the OEC^41^ sports a large variety of stable JT isomers (structures that differ in the relative orientation of their JT axes) at its lower oxidation states,^38^ and in the S1 oxidation state, these favor oxidation leading to distinct redox isomers (structures that differ in the assignment of oxidation states to specific atoms) in the S2, possibly influencing the water oxidation cycle of the OEC.^42^

A detailed investigation of JT and redox isomers of the precatalytic and activated forms of [Mn_4_V_4_O_17_(OAc)_3_]^3–^ at various oxidation states found that JT axes prefer an orientation toward the weaker acetate, water, or OH ligands over the stronger vanadate ligand.^46^ Further, interconversion between redox isomers appears to be associated with low barriers (between 1.5 and 7.6 kcal/mol), while interconversion between JT isomers is almost barrierless (between 0.6 and 1.6 kcal/mol), showing that various redox and JT isomers can be easily accessed in the course of reactions on [Mn_4_V_4_O_17_(OAc)_3_]^3–^.^46^

In this work, we unravel the water oxidation cycle for [Mn_4_V_4_O_17_(OAc)_3_]^3–^ starting from **1**. Density functional theory (DFT) is used to optimize possible intermediates along multiple reaction pathways. A subsequent in-depth sampling of reaction intermediates revealed the multiple roles played by redox isomers and their JT effects in the water oxidation cycle, which introduce a high degree of structural flexibility to the otherwise rigid cubane core. These flexibility effects were further studied by optimizing the minimum energy paths (MEPs) for O−O bond formation including all stationary points, giving a complete picture of this important reaction step. These results are of general importance not only for understanding water oxidation on molecular catalysts but also for advancing the design of Mn-containing WOCs.

## Methods

In the first step, unconstrained geometry optimizations were carried out on guess structures representing possible intermediates of the reaction. As a starting point for the optimization of these guess structures, the activated species **1** was adapted by deleting hydrogen atoms as necessary. The different oxidation states of the reactive oxygen and manganese atoms were specifically targeted by adjusting the multiplicity and charge accordingly. Optimized structures and their Gibbs free energies were obtained using the Gaussian16 package^47^ at the B3LYP/def2-SVP level of theory^48−50^ with Grimme’s D3 dispersion correction.^51^ Solvent effects were accounted for by employing the Polarizable Continuum Model (PCM), an implicit solvation model;^52^ the acetonitrile/H_2_O 9:1 (v/v) solvent composition used in photocatalytic experiments^43,44^ was approximated using the parameters for acetonitrile with a custom epsilon value of 41.589. All calculations were carried out on an all-atom model of the complex using the high-spin configuration.^53−55^ Oxidation states of individual atoms are reported based on their computed Mulliken spin populations.

The results of these initial unconstrained optimizations indicated that both Mn^3+^ and Mn^4+^ centers play a role in the water oxidation mechanism of the cluster. As Mn^3+^ is a d^4^ ion that shows JT distortions in an octahedral coordination environment, these effects were accounted for as follows. For each Mn^3+^ center in a given structure, three orientations of the elongated JT bond axis are possible: in the *x*-, *y*-, or *z*-direction. The global minimum on the potential energy surface is obtained by sampling all three possible JT orientations for each Mn^3+^ center, which results in a large number of distinct isomers.^34,38^ We adopted the sampling procedure of ref (46), wherein individual isomers are obtained from guesses that used constrained preoptimizations; specifically, the two bonds corresponding to the desired JT axis for each Mn^3+^ center are elongated. Additionally, we sampled possible ligand conformers for each protonation state of the cluster. Constrained preoptimizations were carried out using the ORCA 4.2.1 package^56,57^ at the BP86/def2-SVP level of theory,^50,58,59^ with D3 dispersion correction^51^ and PCM (acetonitrile);^52^ the looseopt keyword was employed, as full convergence of these constrained structures was not required. All preoptimized structures were then subjected to an unconstrained optimization to obtain final geometries and energies, again using Gaussian16 at the B3LYP/def2-SVP level of theory. Final JT configurations of the intermediates were determined by comparing the lengths of Mn^3+^–O bonds, with the two longest coaxial bonds indicating the *x*-, *y*-, or *z*-orientation of the JT axis on a given Mn^3+^ center. As in the precatalyst,^46^ not every conceivable redox and JT isomer of an individual intermediate corresponds to a stable minimum on that intermediate’s potential energy surface. In the following, only the most stable isomers for each intermediate in the proposed water oxidation cycle shall be discussed (see Table S1 for a list of all stable isomers and conformers).

Complex **1** and its various reaction intermediates show CS symmetry. No explicit symmetry was employed in our calculations; however, we did not consider symmetry-equivalent redox and JT isomers separately. Furthermore, isomers featuring JT axes oriented toward the vanadate ligand were not specifically targeted, as these had been previously found to be energetically unfavorable.^46^

To study the O–O bond formation, Climbing Image-Nudged Elastic Band (CI-NEB) calculations were carried out using the ORCA 5.0.0 package.^56,57^ We used B3LYP^48,49^ with the def2-SVP basis set,^50,60^ employing D3 dispersion correction^51^ and conductor-like PCM (acetonitrile)^52^ with a custom epsilon value of 41.589 and surface type vdw_gaussian. All stationary points found by NEB calculations were optimized using Gaussian16 at the B3LYP/def2-SVP level of theory, as described above.

To ensure full convergence, the electronic energies of all species discussed herein were refined at the B3LYP/def2-TZVP level of theory. Taken together with thermochemical corrections obtained from frequency calculations at the B3LYP/def2-SVP level of theory at *T* = 298.150 K, we calculated final Gibbs free energies relative to **1** (unless otherwise noted). To account for differing protonation states between structures, an energy correction term is calculated using the approach proposed by Van Voorhis et al.,^7^ wherein the standard free energy of a proton in solution is added for each proton removed from the cluster. To approximate the standard free energy of a proton in the acetonitrile/H_2_O 9:1 (v/v) mixture used in the experiment, we used a 9:1 weighted average of the standard free energy of a proton in acetonitrile (11.0622 eV) and the standard free energy of a proton in water (11.5305 eV),^61^ giving a value of 11.1090 eV for each proton transferred to the solution. A detailed breakdown of the final Gibbs free energies is provided in Table S2.

The thermodynamic limit of water oxidation is defined in this work as the free energy of the reaction (2), computed using B3LYP as 4.56 eV.^62^ According to the Sabatier principle,^63^ a thermodynamically ideal catalyst would overcome this limit in four equal steps, each with a potential of 1.14 eV. Thus, the overpotential of water oxidation using such an ideal catalyst would be entirely kinetic, possibly originating from the reaction barriers of O–O bond formation or O_2_ evolution. In contrast, in a real catalyst, some intermediates are more stabilized than others, resulting in one potential step being larger than the others—the potential-determining step.^6^ The thermodynamic overpotential η of water oxidation is defined as the difference between the potential-determining step of the real catalyst and the step size of an ideal catalyst^14,64^
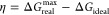
4

## Results and Discussion

The starting point of our study is the activated species [Mn_4_^4+^V_4_O_17_(OAc)_2_(H_2_O)(OH)]^1–^ (**1**) (recall Figure 1b).^45^ As **1** features cofacial H_2_O and OH ligands in close proximity, we assume that water oxidation involves both ligands and their respective metal centers. While a variety of single-center mechanisms could also be imagined, here we focus exclusively on plausible multicenter mechanisms. The two ligands must formally undergo four oxidation and three deprotonation steps, which are assumed to be coupled^6^ (i.e., three proton-coupled electron transfer (PCET) steps and one-electron transfer (ET) step). A priori, the order of these steps is unknown. As proton acceptors are readily available in solution under the photocatalytic conditions used in experiment (acetonitrile/H_2_O 9:1 (v/v), [Ru(bpy)_3_]^2+^, Na_2_S_2_O_8_),^43,44^ deprotonation of the cluster is most likely carried out by the solvent.

Oxidation of the reactive H_2_O and OH ligands could occur in two ways, either intermolecularly by an oxidizing agent present under photocatalytic conditions, i.e., [Ru(bpy)_3_]^3+^, or intramolecularly, with a concomitant reduction of each Mn^4+^ center to Mn^3+^. In the case of intermolecular oxidation, the cluster would serve primarily as a structural support and activator of the ligands, without being directly involved in their redox chemistry. Alternatively, as all Mn centers are in the Mn^4+^ oxidation state in **1**, one can argue that the cluster is storing up to four redox equivalents (much like the OEC),^65^ allowing intramolecular oxidation of the ligands to occur. Both possibilities were accounted for in our calculations for each step of the reaction. This, along with the multiple possible orders of the PCET and ET steps, results in 64 possible pathways for O_2_ formation. These pathways were compared according to the Gibbs free energies of their intermediates, with only the thermodynamically most favorable pathways being discussed here (further results are in Table S1). It should be noted that a recent in situ IR spectroscopy study^66^ has shown that changes of the catalyst under oxidative conditions occur at the Mn centers, while the vanadate ligand remains unaffected. We therefore focus on those redox pathways that involve primarily the Mn_4_O_4_ cubane core and the reactive ligands.

**Figure 2:**
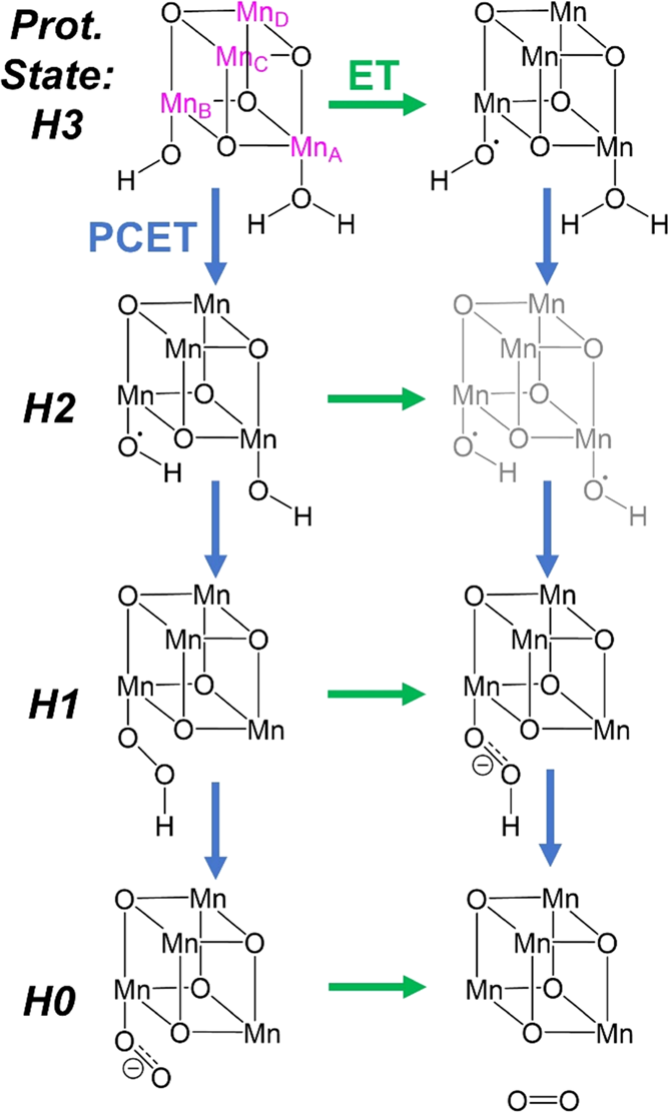
Types of intermediates investigated by direct optimization, starting from **1** (top left) and resulting from the four possible orders of proton-coupled electron transfer (PCET, blue) and electron transfer (ET, green) steps. The protonation state *Hn* (*n* = 0, 1, 2, 3) for each pair of structures is noted on the left. The grayed-out structure could not be optimized, leading to the exclusion of pathways involving such an intermediate from further consideration.

The uncertainty regarding the order of PCET and ET steps results in four possible arrangements of the reaction steps: PCET-PCET-PCET-ET, PCET-PCET-ET-PCET, PCET-ET-PCET-PCET, and ET-PCET-PCET-PCET (see Figure 2). First, we directly optimized intermediates corresponding to each of the structures shown in Figure 2 considering all accessible oxidation states of the Mn centers. The results obtained proved to be pivotal for our further investigation, as will be described next (for a detailed overview of results of these direct optimizations, see Table S1).

We note that the species with two protonated oxyl groups (gray in Figure 2) could not be optimized, indicating that the PCET-ET-PCET-PCET and ET-PCET-PCET-PCET pathways, which necessarily include such a species, may not be feasible. Importantly, the investigation of both intermolecular and intramolecular oxidation for each reaction step showed that intramolecular oxidation of the ligands by the Mn^4+^ centers appears to be thermodynamically favored over intermolecular oxidation, regardless of the order of PCET and ET steps. Finally, our results pointed toward an intramolecular water nucleophilic attack of an OH ligand on the neighboring oxyl radical as a possible mechanism of O–O bond formation, an i-WNA(OH)-type mechanism within the classification proposed by Schilling and Luber.^13^ We therefore expanded our sampling of reaction intermediates to include a greater variety of isomers and conformers.

As redox and JT isomerism were previously shown to play an important role in the reactivity of [Mn_4_V_4_O_17_(OAc)_3_]^3–^,^45,46^ we decided to target the most important isomers and conformers of each intermediate of interest. To this end, we extended the sampling procedure of ref (46) by also targeting multiple ligand arrangements for each redox and JT isomer. Accordingly, we next investigated all four arrangements of reaction steps (PCET-PCET-PCET-ET, PCET-PCET-ET-PCET, PCET-ET-PCET-PCET, and ET-PCET-PCET-PCET) as well as both inter- and intramolecular oxidations. Table 1 gives an overview of which specific redox and JT isomers were targeted at each oxidation state of the cubane core (designated as Mn4444 for Mn_4_^4+^ and Mn3333 for Mn_3_^3+^) and the protonation state of the reactive ligands (designated as *Hn*, where *n* is the number of protons). In total, we investigated 91 unique isomers and 203 individual conformers (considering the various ligand arrangements sampled at each protonation state). A full overview of the resulting 80 optimized geometries can be found in Table S1.

**Table 1:**
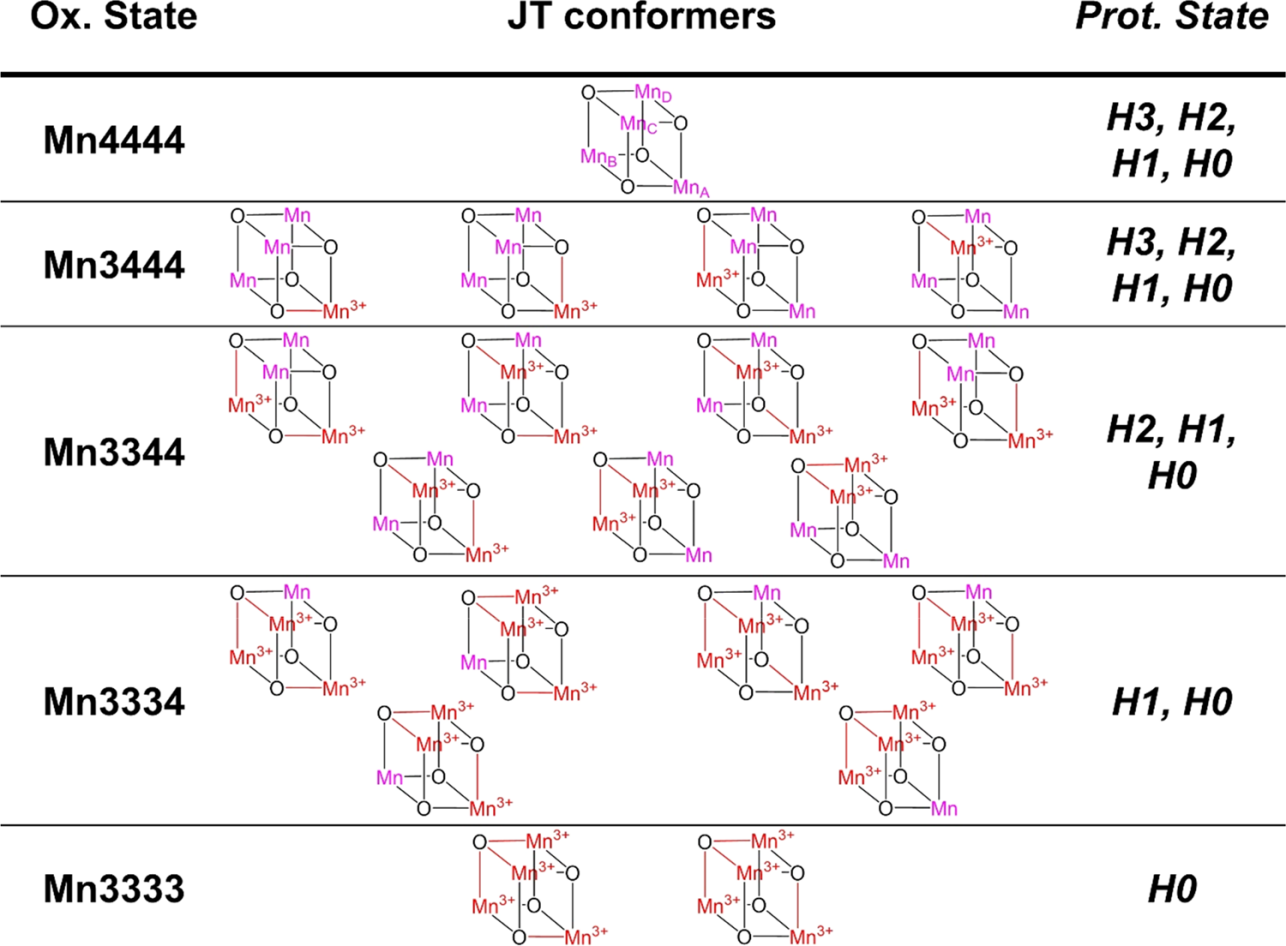
Redox and Jahn–Teller (JT) Isomers Targeted for Sampling at Each Oxidation State^a^

a The nomenclature MnXXXX (left column) indicates the oxidation state of the four Mn atoms, e.g., Mn3333 corresponds to a Mn_4_^3+^ configuration and Mn4444 to a Mn_4_^4+^ configuration of the cubane core. At each oxidation state, a variety of ligand conformations for each relevant protonation state *Hn*, where *n* is the number of protons, were targeted (right column). Mn^3+^ centers and the orientation of their JT axes are marked in red and Mn^4+^ centers in purple.

This extended sampling approach largely confirmed our initial observations. An intermediate with two protonated oxyl groups that would be essential for the ET-PCET-PCET-PCET and PCET-ET-PCET-PCET mechanisms could not be optimized, leading to the conclusion that such an intermediate is too unstable to play any significant role in the water oxidation mechanism of **1**. Furthermore, the energy difference between **1** and the most stable intermediate resulting from a single intermolecular PCET step was found to be 7.50 eV (at the B3LYP/def2-SVP level of theory, see Table S1), which is significantly above the computed thermodynamic limit of water oxidation at 4.56 eV.^62^ It appears therefore that intermolecular ETs are highly unfavorable in the context of the water oxidation cycle of **1**, allowing us to focus exclusively on pathways that feature intramolecular ETs.

**Figure 3:**
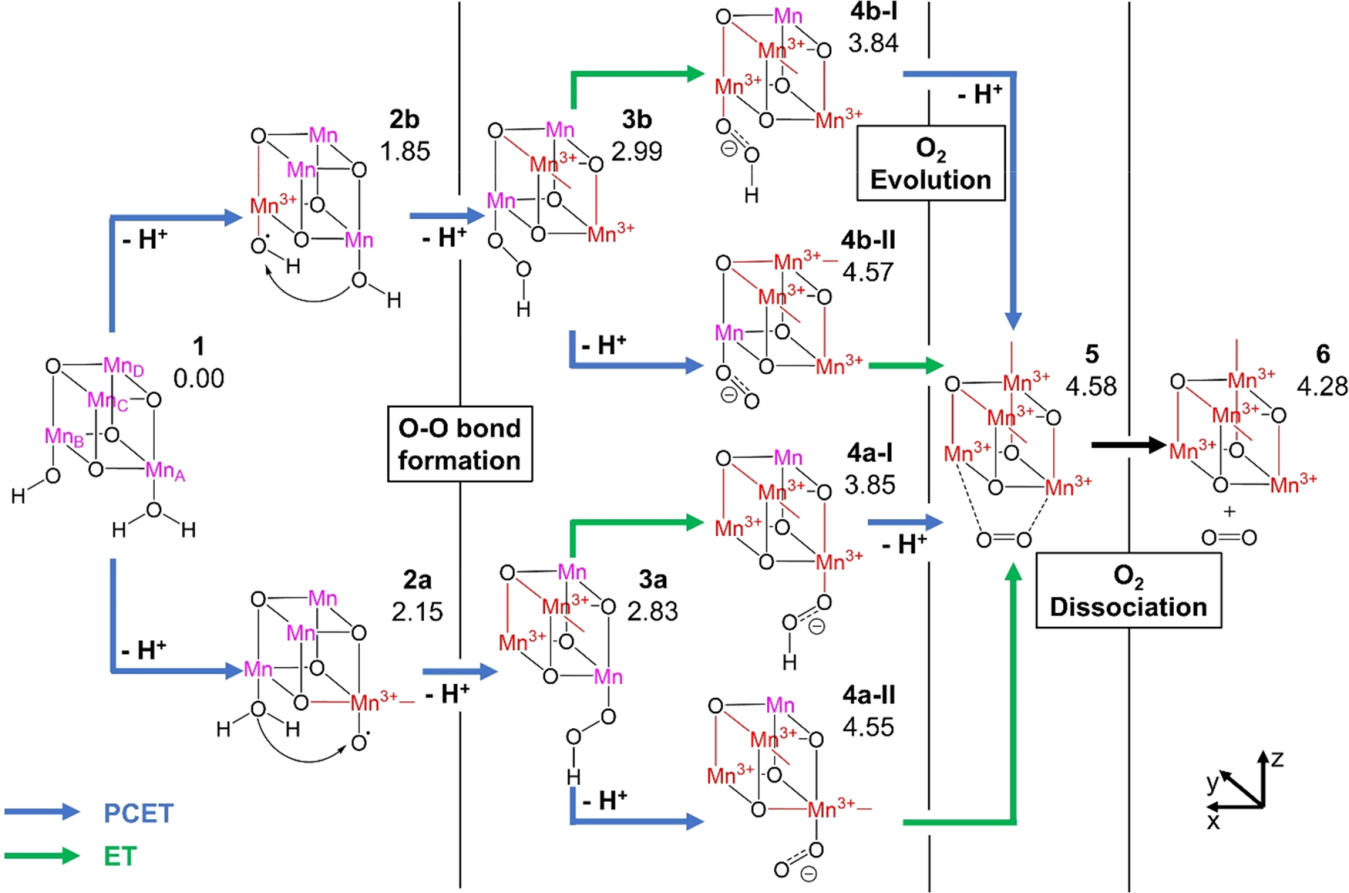
Most stable isomers of intermediates along the thermodynamically most favorable pathways for the PCET-PCET-ET-PCET and PCET-PCET-PCET-ET orders of steps. PCETs are marked by blue arrows, ETs by green arrows, and other reactions by black boxes. Mn^4+^ centers are in purple, and Mn^3+^ centers and their JT axes are in red. Intermediates are labeled **1** through **6**, and their relative Gibbs free energies are presented in eV at the B3LYP/def2-SVP/def2-TZVP level of theory.

The additional sampling of various conformations of the reactive ligands for each intermediate revealed that the nucleophilic attack of OH on the neighboring Mn-oxyl group could take place at either Mn_B_ (Figure 3, top) or Mn_A_ (Figure 3, bottom), with relatively minute differences in the stability of the respective intermediates. Figure 3 shows the most stable isomers found for each intermediate between the activated species **1** and the deactivated catalyst after O_2_ evolution and dissociation **6**.

Starting from **1**, an intramolecular ET from the OH ligand to its Mn_B_^4+^ center, coupled to deprotonation of the neighboring H_2_O ligand, results in intermediate **2b** with a JT axis on Mn_B_^3+^ pointing in the *z*-direction toward the protonated oxyl species, with a reaction energy of 1.85 eV. The next PCET step leads to the formation of the O–O bond (**3b**, 2.99 eV), with some internal rearrangement resulting in Mn_A_^3+^ having a JT axis in the *z*-direction toward an open coordination site and Mn_C_^3+^ having a JT axis in the *y*-direction toward an acetate ligand. The resulting peroxo species is bound to Mn_B_^4+^. Subsequently, an ET step without deprotonation produces **4b-I**, which adds a JT axis on Mn_B_^3+^ in the *z*-direction toward the peroxyl species with a resulting relative energy of 3.84 eV. In contrast, a PCET from **3b** results in **4b-II**, with a new JT axis in the *x*-direction (toward an acetate ligand) on Mn_D_^3+^ and a far higher relative energy of 4.57 eV. In either case, the final redox step leads to the product complex **5** (4.58 eV), in which molecular O_2_ has been formed and is no longer directly bound to any Mn center but loosely associated with the complex (product complex). The species **5** features JT axes on Mn_A_^3+^ and Mn_B_^3+^ in the *z*-direction toward the loosely associated O_2_, on Mn_C_^3+^ in the *y*-direction toward an acetate ligand, and on Mn_D_^3+^ in the *z*-direction toward the vanadate ligand. Dissociation of O_2_ gives the final product **6**, with an identical arrangement of JT axes and a relative energy of 4.28 eV.

Alternatively, an intramolecular PCET at the H_2_O ligand bound to Mn_A_^4+^ results in **2a**, with a proton transfer to the OH ligand yielding an oxyl species bound to Mn_A_^3+^ with a JT axis in the *x*-direction toward a neighboring acetate ligand and a reaction energy of 2.15 eV. The O–O bond formation once again occurs with the next PCET step, resulting in the intermediate **3a** at 2.83 eV. This features JT axes at Mn_B_^3+^ in the *z*-direction toward the open coordination site and at Mn_C_^3+^ in the *y*-direction toward an acetate ligand, with the peroxo species bound to Mn_A_^4+^. Next, either a simple ET results in **4a-I**, with a new JT axis in the *z*-direction on Mn_A_^3+^ toward the peroxyl species (3.85 eV) or a PCET step results in **4a-II**, with a JT axis added in the *x*-direction on Mn_A_^3+^ toward an acetate ligand (4.55 eV). The final steps converge to the product complex **5** and final product **6** as described above.

**Figure 4:**
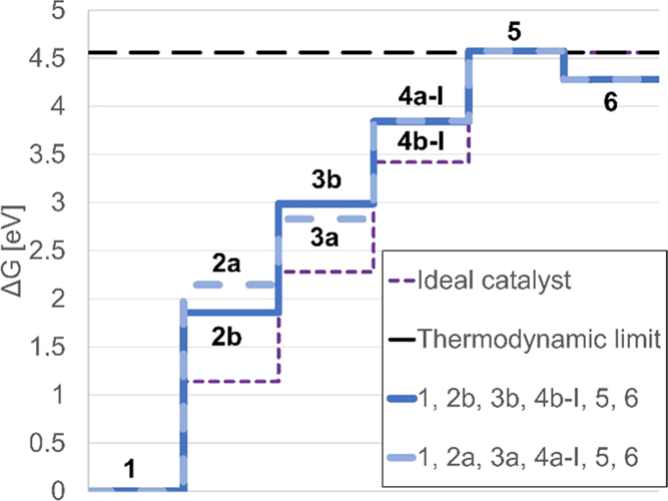
Gibbs free energies of intermediates along the most favorable PCET-PCET-ET-PCET pathways, calculated at the B3LYP/def2-SVP/def2-TZVP level of theory: **1**, **2a**, **3a**, **4a-I**, **5**, **6** (light blue, dashed) vs **1**, **2b**, **3b**, **4b-I**, **5**, **6** (dark blue, continuous). For comparison, the computed thermodynamic limit of water oxidation (black, dashed) and an ideal catalyst with evenly spaced intermediates (purple, dashed).

Comparing the relative energies of the species in Figure 3, it is immediately apparent that out of the intermediates resulting from the penultimate redox step, **4b-I** (3.84 eV) is far more stable than **4b-II** (4.57 eV); the same can be observed for **4a-I** (3.85 eV) versus **4a-II** (4.55 eV). From this, we can conclude that PCET-PCET-ET-PCET is the preferred order of redox steps. To determine whether the formation of the peroxo species is thermodynamically favored to take place on Mn_A_ (pathway: **1**, **2a**, **3a**, **4a-I**, **5**, **6**; light blue in Figure 4) or on Mn_B_ (pathway: **1**, **2b**, **3b**, **4b-I**, **5**, **6**, dark blue in Figure 4), we compare the two resulting pathways to the behavior of an ideal catalyst that overcomes the computed thermodynamic limit of water oxidation (4.56 eV^62^) in four equal steps of 1.14 eV each (purple in Figure 4). The greater the departure from the ideal catalyst, the larger the resulting thermodynamic overpotential, limiting the efficiency of water oxidation by **1**. Figure 4 discloses that the first redox step has the greatest reaction energy—1.85 eV for **2b** and 2.15 eV for **2a**—and therefore differs most significantly from the value of 1.14 eV for an ideal catalyst, making this the potential-determining step. We can thus conclude that the lowest thermodynamic overpotential should result from forming the peroxo species on Mn_B_, following the pathway **1**, **2b**, **3b**, **4b-I**, **5**, **6**. Specifically, the predicted thermodynamic overpotential for this catalytic sequence is 0.71 V, as computed from eq 4.

A closer examination of the intermediates comprising the two most favorable pathways, **1**, **2b**, **3b**, **4b-I**, **5**, **6** and **1**, **2a**, **3a**, **4a-I**, **5**, **6**, reveals the multiple roles that redox isomerizations and JT effects play in enhancing the reactivity of [Mn_4_V_4_O_17_(OAc)_3_]^3–^ (recall Figure 3). First, one can observe that **4b-I** and **4a-I** prominently feature JT axes in the *z*-direction toward the reactive ligand on Mn_B_^3+^ and Mn_A_^3+^, respectively. As JT-distorted bonds break more easily, we can infer that the barrier for the Mn^3+^–O bond cleavage that occurs in the O_2_ evolution step is lowered due to the presence of JT effects in **4b-I** and **4a-I**. Similar behavior has been observed in related OEC model complexes^41^ as well as in the activation of [Mn_4_V_4_O_17_(OAc)_3_]^3–^, where the rate-determining step in the ligand exchange had a lower barrier when a Mn^3+^ center with a JT axis in the direction of the departing ligand was present.^45^

Second, while the redox steps from **3b** via **4b-I** to **5** as well as from **3a** via **4a-I** to **5** require no rearrangement of existing JT axes, but rather involve only the progressive addition of a new JT axis at each emerging Mn^3+^ center, the PCET step from **2b** to **3b** includes significant rearrangement, going from a *z*-axis on Mn_B_^3+^ to a *z*-axis on Mn_A_^3+^ and a *y*-axis on Mn_C_^3+^. Similarly, the PCET step from **2a** to **3a** involves going from an x-axis on Mn_A_^3+^ to a *z*-axis on Mn_B_^3+^ and a *y*-axis on Mn_C_^3+^. As these steps are key to the O–O bond formation, this seemingly inexplicable behavior awoke our curiosity.

The reaction from **2b** to **3b** comprises at least three elementary steps: deprotonation, ET, and O–O bond formation. As the nucleophilic attack by the OH ligand on the Mn_B_^3+^-oxyl cannot take place as long as the oxyl is protonated, we can safely assume that deprotonation must occur first. We therefore optimized a guess structure based on **2b**, from which the proton on the oxyl ligand has been removed (**OO1**). Next, a CI-NEB calculation was carried out between **OO1** and **3b** to ascertain the order of ET and O–O bond formation steps along the MEP. Figure S1 shows the relative energy along the MEP and pairwise atom distances.

**Figure 5:**
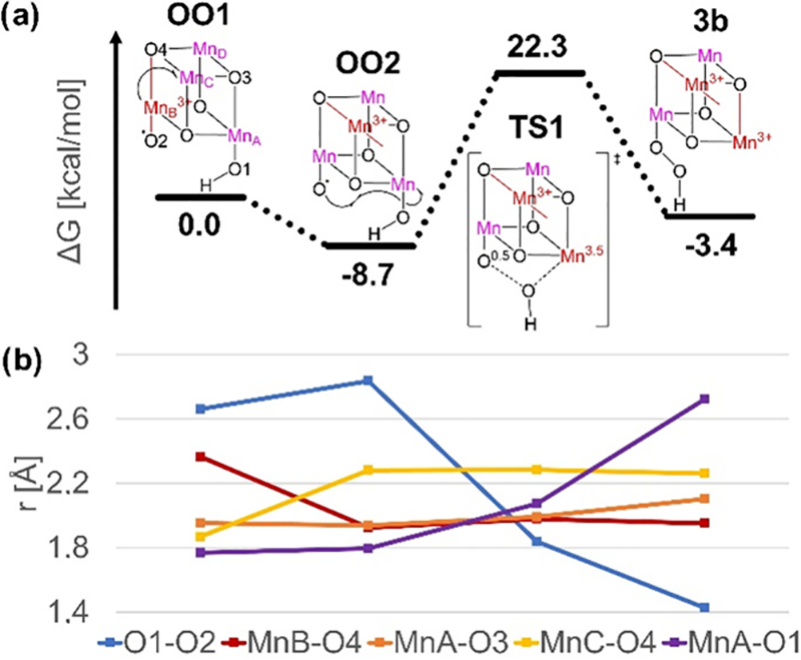
(a) Stationary points along the MEP for O–O bond formation and their relative energies in kcal/mol. (b) Bond lengths for stationary points along the MEP; O1–O2 in blue, Mn_B_–O4 along the *z*-axis in red, Mn_A_–O3 along the *z*-axis in orange, Mn_C_–O4 along the *y*-axis in yellow, and Mn_A_–O1 in purple.

The NEB calculation detected two stationary points along the MEP for O–O bond formation that we subsequently optimized (Figure 5a and Tables S3 and S4). Starting from **OO1**, an ET from Mn_B_ to Mn_C_ results in a stable intermediate (**OO2**, −8.7 kcal/mol, *r*_O1–O2_ = 2.84 Å) that is a redox isomer of **OO1**, featuring a JT axis in the *y*-direction along Mn_C_^3+^–O4 as well as a more electrophilic Mn_B_^4+^-oxyl group (see also Figure 5b, red and yellow). From **OO2**, the expected nucleophilic attack of OH at Mn_B_^4+^-oxyl proceeds via a barrier of 31.0 kcal/mol, which compares favorably to the water nucleophilic attack barriers previously described for related WOCs.^24−26^ The associated transition state (**TS1**) has the attacking OH ligand suspended halfway between its metal center (*r*_MnA–O1_ = 2.07 Å) and the oxyl ligand (*r*_O1–O2_ = 1.84 Å) and features spin populations of 3.56 on Mn_A_ and 0.53 on O2. This surprising result indicates that O–O bond formation occurs by homolytic cleavage of the Mn_A_–O1 bond, resulting in a one-electron reduction of Mn_A_^4+^ to Mn_A_^3+^ and radical coupling between O1 and O2. As can be seen in Figure 5b, the formation of the O1–O2 bond (blue), the cleavage of the Mn_A_–O1 bond (purple), and the ET to Mn_A_ (shown by the eventual emergence of JT distortions in the *z*-direction along Mn_A_–O3, orange) are clearly coupled. Inspection of the normal mode corresponding to the imaginary frequency of **TS1** confirms that all three processes are in fact concerted. The product **3b** has an additional JT axis on Mn_A_^3+^ in the *z*-direction toward the now-open coordination site, with the resulting peroxide species (*r*_O–O_ = 1.43 Å) bound to Mn_B_^4+^, giving an overall reaction energy of −3.4 kcal/mol for O–O bond formation along the **OO1** to **3b** pathway.

We also investigated the alternative O–O bond formation pathway between **2a** and **3a** by first optimizing a deprotonated guess structure based on **2a** (**OO3**). A NEB calculation between **OO3** and **3a** was carried out and is shown in Figure S2, with broadly similar results to the NEB calculation between **OO1** and **3b** described above: A redox isomerization leads to an intermediate **OO4** (−6.1 kcal/mol, *r*_O1–O2_ = 2.83 Å), featuring a JT axis in the *y*-direction along Mn_C_^3+^–O4 as well as a more electrophilic Mn_A_^4+^-oxyl group. Unfortunately, the transition state between **OO4** and **3a** could not be optimized, but the barrier is expected to be comparable to that presented by **TS1** (see above).

Finally, as **3a** is 3.6 kcal/mol more stable than **3b**, a conversion of **3b** to **3a** via migration of the peroxo species from Mn_B_ to Mn_A_ with concomitant redox isomerization would be thermodynamically favorable. A NEB calculation revealed that this is a simple one-step reaction with a single transition state, which was subsequently optimized as **TS3** (see Figure S3). The barrier for conversion from **3a** to **3b** was found to be quite low, only 6.7 kcal/mol, making this process feasible and opening up the possibility of a crossover pathway consisting of **1**, **2b**, **3b**, **3a**, **4a-I**, **5**, **6**.

We thus conclude that structural flexibility in the form of facile redox and JT isomerizations plays an essential role in enhancing the reactivity of the [Mn_4_V_4_O_17_(OAc)_3_]^3–^ WOC not only during the activation^45^ but also in the water oxidation cycle itself. A weak, JT-distorted bond contributes to lowering the barrier for O_2_ evolution, and a redox isomerization of **OO1** with its Mn_B_^3+^-oxyl yields **OO2**, which features a more electrophilic Mn_B_^4+^-oxyl group. This redox isomerization must occur first to enable O–O bond formation via attack by a neighboring OH ligand, and that attack takes place in concert with an ET to Mn_A_ and the emergence of a new JT axis at that metal center. Our observations are reminiscent of the behavior of the OEC recently described by Drosou et al.,^42^ who found that different JT isomers in the S1 state influence ligand exchange kinetics as well as favor the formation of distinct redox isomers in the S2, thereby directly influencing the mechanism of water oxidation in the natural system. It should be noted, however, that the high catalytic activity of the OEC relies intimately on the protein environment in which it is embedded, while our calculations deal with [Mn_4_V_4_O_17_(OAc)_3_]^3–^ in solution. It would therefore be interesting to see whether the integration of [Mn_4_V_4_O_17_(OAc)_3_]^3–^ into a functionalized soft matter matrix would further increase its activity.

## Conclusions

In this paper, we propose a water oxidation mechanism for the bioinspired water oxidation catalyst [Mn_4_V_4_O_17_(OAc)_3_]^3–^, starting from the activated species [Mn_4_^4+^V_4_O_17_(OAc)_2_(H_2_O)(OH)]^1–^ (**1**). After activation, the catalyst holds four redox equivalents in the form of four Mn^4+^ centers; the catalyst also binds cofacial OH and H_2_O ligands that are positioned in close proximity, allowing them both to participate in water oxidation. Water oxidation proceeds by an intramolecular ET from the OH ligand of Mn_B_ to that metal center, coupled with deprotonation of the neighboring H_2_O ligand. This is followed by another such PCET and O–O bond formation, with a peroxo species formed on Mn_B_. An intramolecular ET and final PCET lead to the evolution and subsequent dissociation of O_2_, resulting in the deactivated catalytic species [Mn_4_^3+^V_4_O_17_(OAc)_2_]^4–^ with one open coordination site on Mn_A_ and Mn_B_ each.

While the presence of JT axes has been noted in OEC models and carefully analyzed to allow for a better comparison with experimental X-ray structures,^32−42^ here we investigate the explicit influence of these distortions on the water oxidation cycle. We found that redox isomerism and JT effects play a prominent role in the water oxidation cycle of [Mn_4_V_4_O_17_(OAc)_3_]^3–^: In the O_2_ release step, JT distortions contribute to lowering the barrier of Mn_B_^3+^–O bond cleavage in a straightforward manner. The formation of the O–O bond is preceded by a redox isomerization step and appears to be concerted with an ET from the reactive ligands to Mn_A_ and the emergence of a new JT axis at that metal center. These findings are of particular interest when one considers the role played by redox isomerism and JT effects in the natural OEC; there, JT effects were shown to influence both ligand exchange kinetics and water oxidation pathways.^41,42^ As redox isomerism and JT effects are present in cubane structures with Mn^3+^/Mn^4+^ centers, we argue that these flexibility-enhancing effects should be harnessed in the design of novel bioinspired WOCs. Future work on [Mn_4_V_4_O_17_(OAc)_3_]^3–^ will focus on the stability of the catalyst, with special attention paid to its regeneration, degradation, and modes of integration in soft matter matrices.

## Supporting Information Available

Molecular structures, Mulliken spin populations, electronic energies, thermochemical contributions, free energies, and nudged elastic band results (PDF)
