# Supplementary material for: Flexibility Enhances Reactivity: Redox Isomerism and Jahn–Teller Effects in a Bioinspired Mn4O4 Cubane Water Oxidation Catalyst
Source: ACS Catal. 2021 Oct 18;11(21):13320–9. doi: 10.1021/acscatal.1c03566 (PMC8576808; doi:10.1021/acscatal.1c03566)
Supplement: Supplementary file 1 — cs1c03566_si_001.pdf [file cs1c03566_si_001.pdf]

Supporting Information:

Flexibility Enhances Reactivity: Redox Isomerism and Jahn-Teller Effects in a Bioinspired Mn_4O_4 Cubane Water Oxidation Catalyst

Ludwig Schwiedrzik,[a,*] Vera Brieskorn,[a] Leticia González[a,*]

[a] Institute of Theoretical Chemistry, Faculty of Chemistry, University of Vienna, Währinger Straße 17, 1090 Vienna, Austria

[*] ludwig.schwiedrzik@univie.ac.at, leticia.gonzalez@univie.ac.at

Contents

I.	List of all stable isomers and conformers	S2
II.	Detailed energy breakdown, named Intermediates	S5
III.	Nudged elastic band calculations	S6
IV.	List of O-O bond formation stationary points	S7
V.	Detailed energy breakdown, O-O bond formation stationary points	S8
VI.	Cartesian coordinates of selected intermediates, transition states	S9

I. List of all stable isomers and conformers

Table S1: Unique optimized isomers and conformers of possible intermediates in the water oxidation cycle of $[Mn_4V_4O_{17}(OAc)_3]^{3-}$, grouped according to the overall redox state of the cubane core (e.g Mnx444, corresponding to a $Mn^{4+}{}_3Mn^{3+}$ configuration with a JT axis in x direction on Mn_A), charge q and multiplicity 2S+1. Columns from left to right: Redox and JT configuration of the cubane; configuration of reactive ligands on Mn_B and Mn_A; Gibbs free energy relative to **1**, computed at the B3LYP/def2-SVP level of theory (in eV); spin populations of $Mn_{A\text{-}D}$ and of the oxygen atoms of the reactive ligands, where O2 is bound to Mn_B and O1 is bound to Mn_A in **1**; $r_{O\text{-}O}$ distance between O1 and O2 atoms (in Å). Further notes: named intermediates in bold, †=structure obtained by direct optimization, ‡=no stable intermediates found.

**Cubane config**	**Ligands**		**$\Delta G_{DZ,rel}$ [eV]**	**Spin populations**						**$r_{O\text{-}O}$ [Å]**	**Notes**
	**Mn_B-**	**Mn_A-**		**Mn_A**	**Mn_B**	**Mn_C**	**Mn_D**	**O2**	**O1**		
**Mn4444**	**q=-1**		**2S+1=13**								
Mn4444	OH	OH_2	0.00	3.05	2.93	2.93	2.92	0.01	0.00	2.61	†,**1**
Mn4444	OH_2	OH	0.04	3.00	2.94	2.93	2.92	-0.01	0.05	2.62	
**Mn4444**	**q=0**		**2S+1=14**								
Mn4444	O*H	OH_2	7.69	3.04	2.85	2.88	2.88	0.93	-0.01	2.88	
Mn4444	OH_2	O*H	7.74	3.00	2.93	2.87	2.87	-0.01	0.98	2.81	†
**Mn4444**	**q=-1**		**2S+1=14**								
Mn4444	O*	OH_2	7.50	3.05	2.83	2.92	2.92	1.01	-0.01	2.68	†
Mn4444	O*H	OH	7.63	3.02	2.86	2.92	2.92	0.98	0.02	2.51	
Mn4444	OH	O*H	7.65	3.00	2.93	2.92	2.92	0.00	0.99	2.50	
**Mn4444**	**q=0**		**2S+1=15**								‡
**Mn4444**	**q=-1**		**2S+1=13**								
Mn4444	OOH		13.89	3.12	2.96	2.92	2.92	-0.04	-0.01	1.42	†
Mn4444		OOH	13.96	3.00	2.99	2.91	2.91	0.03	0.01	1.43	
**Mn4444**	**q=0**		**2S+1=14**								
Mn4444	OO*H		20.34	3.11	2.91	2.87	2.87	0.62	0.36	1.30	†
Mn4444		OO*H	20.35	2.86	3.03	3.00	2.86	0.38	0.62	1.32	
**Mn4444**	**q=-1**		**2S+1=14**								
Mn4444	OO*		19.68	3.11	2.99	2.91	2.91	0.40	0.49	1.28	†
**Mn4444**	**q=0**		**2S+1=15**								
Mn4444	O_2		25.58	3.13	3.00	2.86	2.86	1.00	1.00	1.20	†
**Mn3444**	**q=-1**		**2S+1=15**								
Mn44y4	O*H	OH_2	1.46	3.03	2.92	3.86	2.96	0.95	-0.01	2.82	
Mn44y4	OH_2	O*H	1.48	2.99	2.97	3.86	2.96	-0.01	0.96	2.77	
Mn4z44	OH_2	O*H	1.50	3.00	3.87	2.96	2.95	0.02	0.97	2.67	
Mn4z44	O*H	OH_2	1.51	3.04	3.85	2.96	2.95	1.03	0.00	2.82	†
Mnz444	O*H	OH_2	1.78	3.92	2.88	2.96	2.97	0.87	0.02	3.27	
Mnx444	O*H	OH_2	1.78	3.92	2.94	2.97	2.95	0.71	0.00	3.82	
Mnz444	OH_2	O*H	1.83	3.90	2.97	2.95	2.96	-0.01	1.02	2.80	
Mnx444	OH_2	O*H	1.89	3.88	2.97	2.93	2.95	-0.01	0.73	2.81	
**Mn3444**	**q=-2**		**2S+1=15**								

Mn4z44	O*H	OH	1.85	3.03	3.86	2.99	2.99	1.04	0.03	2.57	†,**2b**
Mnx444	O*	OH_2	2.02	3.91	2.92	2.99	2.98	1.00	-0.01	2.65	
Mnx444	OH_2	O*H	2.15	3.87	3.01	2.98	2.98	-0.03	1.01	2.67	**2a**
Mnz444	OH	O*H	2.20	3.89	2.98	3.00	2.99	0.00	1.04	2.55	
Mnz444	OH	O*H	2.31	3.90	2.97	3.00	2.99	0.30	0.75	2.22	
**Mn3444**	**q=–1**	**2S+1=16**									‡
**Mn3444**	**q=–2**	**2S+1=14**									
Mn4z44		OOH	7.70	3.05	3.89	2.99	2.99	-0.01	0.01	1.42	
Mnz444	OOH		7.97	3.92	3.04	3.00	3.00	-0.06	-0.01	1.42	†
Mn44y4		OOH	8.09	3.03	3.03	3.88	2.99	0.00	0.00	1.44	
Mn44y4	OOH		8.14	3.07	2.98	3.86	2.99	-0.01	-0.01	1.44	
Mn4z44	OOH		8.41	3.09	3.82	2.99	2.99	0.07	-0.02	1.45	
**Mn3444**	**q=–1**	**2S+1=15**									
Mn4z44		OO*H	13.74	3.03	3.90	2.95	2.95	0.63	0.36	1.30	
Mn44y4		OO*H	14.00	3.03	3.03	3.86	2.95	0.64	0.35	1.30	
Mnz444	OO*H		14.01	3.93	2.94	2.95	2.95	0.63	0.34	1.30	
Mn4z44	OO*H		14.02	3.10	3.86	2.95	2.94	0.70	0.33	1.31	†
Mn44y4	OO*H		14.03	3.08	2.96	3.86	2.95	0.64	0.34	1.30	
**Mn3444**	**q=–2**	**2S+1=15**									
Mn4z44		OO*	24.62	3.16	3.88	2.98	2.98	0.30	0.59	1.29	†
Mnz444	OO*		24.78	3.92	3.28	3.00	3.00	0.46	0.21	1.26	
Mn44y4	OO*		24.89	3.07	2.99	3.87	2.98	0.48	0.49	1.29	
**Mn3444**	**q=–1**	**2S+1=16**									
Mn4z44	O_2		29.97	3.10	3.90	2.94	2.94	1.00	1.00	1.20	†
Mn44y4	O_2		30.20	3.09	3.02	3.86	2.94	1.00	1.00	1.20	
Mnz444	O_2		30.36	3.94	2.98	2.94	2.94	0.91	1.06	1.20	
**Mn3344**	**q=–2**	**2S+1=17**									‡
**Mn3344**	**q=–3**	**2S+1=15**									
Mn4zy4		OOH	2.93	3.06	3.87	3.87	3.01	-0.01	0.01	1.43	**3a**
Mnz4y4	OOH		3.08	3.91	3.06	3.90	3.03	-0.03	0.00	1.43	†,**3b**
Mnxz44		OOH	3.12	3.93	3.94	3.03	3.04	-0.05	0.00	1.43	
Mn44yx		OOH	3.19	3.06	2.99	3.89	3.88	-0.01	0.00	1.44	
Mn44yx	OOH		3.46	3.06	2.99	3.86	3.86	0.00	-0.01	1.46	
Mnzz44	OOH		3.58	3.92	3.83	3.05	3.05	0.11	0.04	1.47	
Mn4zy4	OOH		3.61	3.08	3.83	3.87	2.99	0.07	-0.02	1.46	
**Mn3344**	**q=–2**	**2S+1=16**									
Mnzz44	OO*H		8.36	3.93	3.88	3.02	3.02	0.71	0.32	1.31	†
Mn4zy4		OO*H	8.43	3.02	3.88	3.86	2.98	0.65	0.34	1.31	
Mnz4y4	OO*H		8.51	3.91	2.99	3.90	3.01	0.66	0.33	1.31	
Mn44yx		OO*H	8.65	3.03	2.98	3.87	3.86	0.65	0.34	1.30	
Mn4zy4	OO*H		8.68	3.07	3.85	3.85	2.98	0.71	0.31	1.31	

Mn44yx	OOH*		8.70	3.07	2.95	3.86	3.86	0.67	0.32	1.31	
Mnz4y4		OO*H	8.76	3.89	3.04	3.90	3.01	0.71	0.32	1.31	
**Mn3344**	**q=-3**	**2S+1=16**									
Mn4zy4		OO*	8.66	3.13	3.87	3.87	3.00	0.59	0.33	1.29	
Mnz4y4	OO*		8.77	3.90	3.10	3.90	3.04	0.57	0.35	1.29	
Mn44yx		OO*	8.85	3.07	2.98	3.87	3.87	0.44	0.52	1.30	
Mnxz44		OO*	8.91	4.01	3.93	3.03	3.03	0.60	0.28	1.30	
Mnzz44	OO*		8.98	3.89	3.86	3.04	3.04	0.57	0.57	1.31	†
**Mn3344**	**q=-2**	**2S+1=17**									
Mnzz44	O_2		13.19	3.93	3.90	3.01	3.01	1.00	1.00	1.20	†
Mn4zy4	O_2		13.46	3.07	3.88	3.85	2.98	1.00	1.00	1.20	
Mnz4y4	O_2		13.53	3.92	3.03	3.90	3.01	1.00	1.00	1.20	
Mn44yx	O_2		13.63	3.07	2.97	3.86	3.86	1.00	1.00	1.20	
**Mn3334**	**q=-3**	**2S+1=17**									
Mnzzy4	OO*H		3.89	3.91	3.87	3.89	3.03	0.71	0.31	1.31	†,**4b-I**
Mnzzy4		OO*H	3.90	3.89	3.89	3.89	3.03	0.71	0.31	1.31	**4a-I**
Mnz4yx	OO*H		3.93	3.91	2.98	3.90	3.90	0.67	0.32	1.31	
Mn4zyz		OO*H	4.15	2.99	3.86	3.86	3.90	0.66	0.32	1.31	
**Mn3334**	**q=-4**	**2S+1=17**									
Mnz4yx	OO*		4.73	3.89	3.04	3.90	3.90	0.41	0.58	1.30	**4b-II**
Mnxzy4		OO*	4.81	3.97	3.91	3.90	3.05	0.61	0.32	1.31	**4a-II**
Mn4zyz		OO*	4.84	3.12	3.86	3.87	3.89	0.58	0.32	1.29	
Mnzzy4		OO*	4.91	3.89	3.87	3.90	3.03	0.56	0.57	1.32	†
Mn4zyx		OO*	4.98	3.14	3.82	3.84	3.84	0.58	0.33	1.29	
Mn4zyy		OO*	5.05	3.08	3.86	3.87	3.89	0.59	0.36	1.30	
Mnzxy4	OO*		5.06	3.92	3.98	3.90	3.07	0.32	0.60	1.30	
**Mn3334**	**q=-3**	**2S+1=18**									
Mnzzy4	O_2		8.61	3.91	3.89	3.89	3.03	1.00	1.00	1.20	†
Mnz4yx	O_2		8.89	3.91	2.96	3.90	3.90	0.95	1.03	1.20	
**Mn3333**	**q=-4**	**2S+1=19**									
Mnzzyz	O_2		4.91	3.90	3.86	3.89	3.89	1.00	1.00	1.20	†,**5**
**Mn3333**	**q=-4**	**2S+1=19**									
Mnzzyz	O_2 ($r = \infty$)		4.70	3.90	3.87	3.89	3.88				†,**6**

II. Detailed energy breakdown, named Intermediates

Table S2: Named intermediates in the water oxidation cycle of $[Mn_4V_4O_{17}(OAc)_3]^{3-}$, with detailed breakdown of electronic and thermochemical contributions to the Gibbs free energy. Columns from left to right: Redox and JT configuration of the cubane; configuration of reactive ligands on Mn_B and Mn_A; electronic energy relative to **1** at the B3LYP/def2-SVP level of theory; electronic energy relative to **1** at the B3LYP/def2-SVP/def2-TZVP level of theory; thermal correction to Enthalpy; thermal correction to Entropy; Grimme-D3 dispersion energy; energy correction for protons transferred to solution; final Gibbs free energy relative to **1**, computed at the B3LYP/def2-SVP/def2-TZVP level of theory. All energy values are given in eV. Further notes: named intermediates in bold.

Cubane config	Ligands		$\Delta E_{DZ,rel}$ [eV]	$\Delta E_{TZ,rel}$ [eV]	ΔH [eV]	ΔS [eV]	$\Delta E_{D3,corr}$ [eV]	ΔG^{H+}_{corr} [eV]	$\Delta G_{TZ,rel}$ [eV]	Notes
	Mn_B-	Mn_A-								
Mn4444	OH	OH_2	-3.91	-3.91	6.90	0.0100	-2.36	0.0000	0.00	**1**
Mnx444	OH_2	O*H	9.74	9.74	6.55	0.0102	-2.29	11.1090	2.15	**2a**
Mn4z44	O*H	OH	9.50	9.50	6.50	0.0102	-2.28	11.1090	1.85	**2b**
Mn4zy4		OOH	21.93	21.83	6.26	0.0102	-2.18	22.2181	2.83	**3a**
Mnz4y4	OOH		22.10	22.00	6.26	0.0102	-2.17	22.2181	2.99	**3b**
Mnzzy4		OO*H	23.06	23.01	6.24	0.0107	-2.08	22.2181	3.85	**4a-I**
Mnzzy4	OO*H		23.07	23.02	6.24	0.0107	-2.08	22.2181	3.84	**4b-I**
Mnxzy4		OO*	35.34	35.08	5.90	0.0104	-2.07	33.3271	4.55	**4a-II**
Mnz4yx	OO*		35.24	35.08	5.90	0.0103	-2.08	33.3271	4.57	**4b-II**
Mnzzyz	O_2		35.65	35.32	5.90	0.0111	-2.01	33.3271	4.58	**5**
Mnzzyz	O_2 (r = ∞)		35.78	35.36	5.86	0.0121	-1.94	33.3271	4.28	**6**

Final Gibbs free energies are obtained according to

$$\Delta G_{TZ,rel}(i) = \left(\Delta E_{TZ}(i) + \Delta H(i) - T\Delta S(i) - \Delta G^{H+}_{corr}(i)\right) - \Delta G_{TZ}(\mathbf{1}),$$

wherein $\Delta G_{TZ,rel}(i)$ is the Gibbs free energy of species *i* at the B3LYP/def2-SVP/def2-TZVP level of theory relative to $\Delta G_{TZ}(\mathbf{1})$, $\Delta E_{TZ}(i)$ is the electronic energy of species *i* at the B3LYP/def2-TZVP level of theory, $\Delta H(i)$ is the thermal correction of the enthalpy for species *i* obtained from frequency calculations at the B3LYP/def2-SVP level of theory, T = 298.150 K, $\Delta S(i)$ is the thermal correction to the entropy for species *i* obtained from frequency calculations at the B3LYP/def2-SVP level of theory, and $\Delta G^{H+}_{corr}(i)$ is the correction to the Gibbs energy arising from protons transferred to solution for species *i*. For simplicity, here we define

$$\Delta E_{TZ,rel}(i) = \Delta E_{TZ}(i) - \Delta G_{TZ,rel}(\mathbf{1}).$$

It should be noted that differences in Gibbs free energy between intermediates arise primarily from the difference in electronic energy (see Table S2). There is some small variance in enthalpic and entropic contributions that we ascribe to the differing number of protons between intermediates and is effectively compensated by the correction term for protons transferred to solution ΔG^{H+}_{corr}.

III. Nudged elastic band calculations

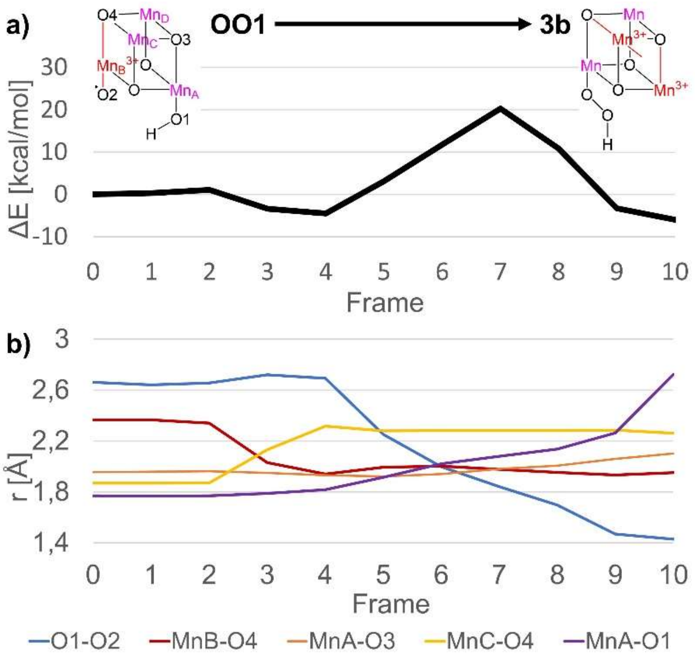

Figure S1: a) Energy changes along the minimum energy path (MEP) between **OO1** and **3b**. b) Bond lengths along the MEP between **OO1** and **3b**; O1-O2 in blue, Mn_B-O4 along z-axis in red, Mn_A-O3 along z-axis in orange, Mn_C-O4 along y-axis in yellow, and Mn_A-O1 in purple.

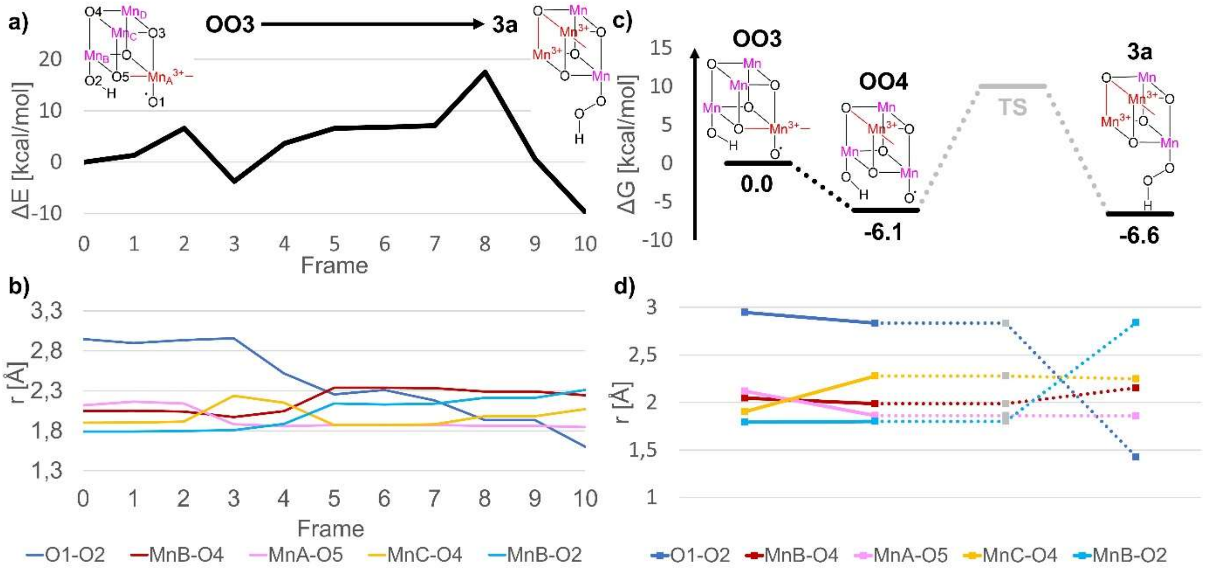

Figure S2: a) Energy changes along the minimum energy path (MEP) between **OO3** and **3a** (convergence: max(Fp)=0.02, RMS(FP)=0.01, max(FCI)=0.05, RMS(FCI)=0.002). b) Bond lengths along the MEP between **OO3** and **3a**; O1-O2 in blue, Mn_B-O4 along z-axis in red, Mn_A-O5 along x-axis in pink, Mn_C-O4 along y-axis in yellow, and Mn_B-O2 in cyan. c) Stationary points along the MEP for conversion of **3b** to **3a** and their relative energies in kcal/mol (TS could not be optimized, shown in grey). d) Bond lengths for stationary points along the MEP; color scheme as in b; TS could not be optimized, shown in grey).

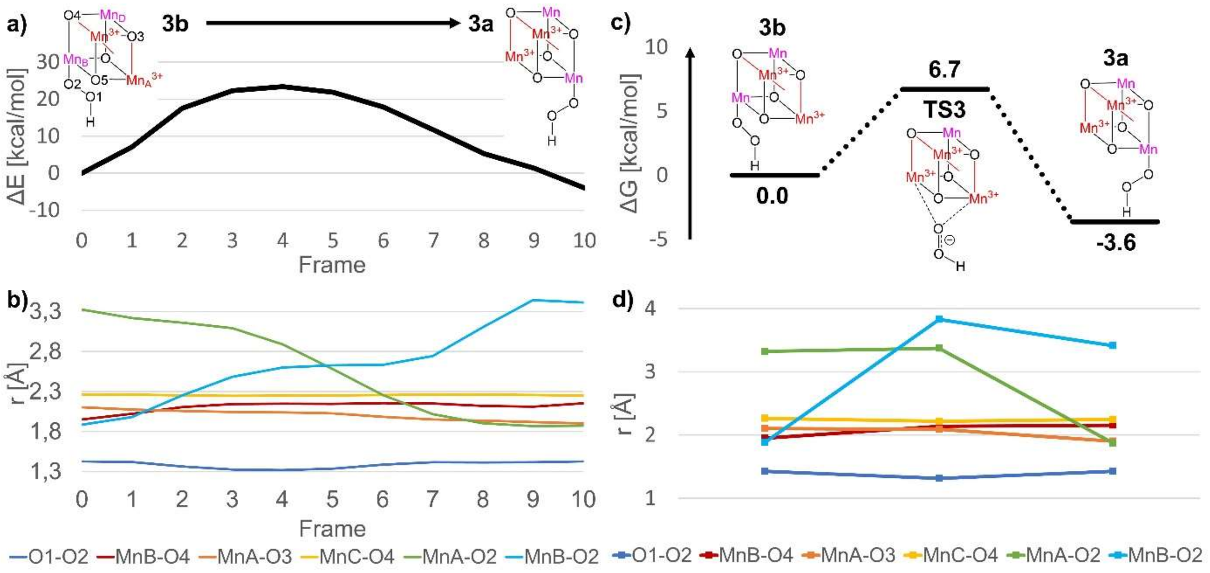

Figure S3: a) Energy changes along the minimum energy path (MEP) between **3b** and **3a**. b) Bond lengths along the MEP between **3b** and **3a**; O1-O2 in blue, Mn_B-O4 along z-axis in red, Mn_A-O3 along z-axis in orange, Mn_C-O4 along y-axis in yellow, and Mn_A-O2 in green, Mn_B-O2 in cyan. c) Stationary points along the MEP for conversion of **3b** to **3a** and their relative energies in kcal/mol. d) Bond lengths for stationary points along the MEP; color scheme as in b).

IV. List of O-O bond formation stationary points

Table S3: Stationary points along the MEPs for O-O bond formation. Columns from left to right: Redox and JT configuration of the cubane (e.g Mnx444, corresponding to a $Mn^{4+}{}_3Mn^{3+}$ configuration with a JT axis in x direction on Mn_A); configuration of reactive ligands on Mn_B and Mn_A; Gibbs free energy relative to **1**, computed at the B3LYP/def2-SVP/def2-TZVP level of theory (in eV); spin populations of $Mn_{A\text{-}D}$ as well as the oxygen atoms of the reactive ligands, where O2 is bound to Mn_B and O1 is bound to Mn_A in **1**; O1-O2 distance in Å. Further notes: names of intermediates and TSs in bold.

**Cubane redox state**	**Ligands**		**$\Delta G_{TZ,rel}$ [eV]**	**Spin populations**						**$r_{O\text{-}O}$ [Å]**	**Notes**
	**Mn_B-**	**Mn_A-**		**Mn_A**	**Mn_B**	**Mn_C**	**Mn_D**	**O2**	**O1**		
Mn4z44	O*	OH	3.14	2.95	3.81	3.03	3.03	1.14	0.10	2.66	**OO1**
Mn44y4	O*	OH	2.76	2.98	2.92	3.88	3.02	1.03	0.08	2.84	**OO2**
Mn44y4	$O^{0.5}$	OH	4.10	3.56	2.95	3.89	3.03	0.53	0.01	1.84	**TS1**
Mnz4y4	OOH		2.99	3.91	3.05	3.90	3.03	-0.03	0.01	1.43	**3b**
Mnx444	OH	O*	3.12	3.86	2.98	3.01	3.01	0.04	1.01	2.95	**OO3**
Mn44y4	OH	O*	2.85	2.96	2.95	3.87	3.01	0.07	1.04	2.83	**OO4**
Mn4zy4		OOH	2.83	3.06	3.87	3.87	3.01	-0.01	0.01	1.43	**3a**
Mnzzy4	-OO*H-		3.41	3.91	3.89	3.89	3.02	-0.66	-0.33	1.32	**TS3**

V. Detailed energy breakdown, O-O bond formation stationary points

Table S4: Named intermediates and transition states along the MEPs of O-O bond formation in $[Mn_4V_4O_{17}(OAc)_3]^{3-}$, with detailed breakdown of electronic and thermochemical contributions to the Gibbs free energy. Columns from left to right: Redox and JT configuration of the cubane; configuration of reactive ligands on Mn_B and Mn_A; electronic energy relative to **1** at the B3LYP/def2-SVP level of theory; electronic energy relative to **1** at the B3LYP/def2-SVP/def2-TZVP level of theory; thermal correction to enthalpy; thermal correction to entropy; Grimme-D3 dispersion energy; energy correction for protons transferred to solution; final Gibbs free energy relative to **1** at the B3LYP/def2-SVP/def2-TZVP level of theory. All energy values are given in eV. Further notes: named intermediates, transition states in bold.

**Cubane config**	**Ligands**		**$\Delta E_{DZ,rel}$ [eV]**	**$\Delta E_{TZ,rel}$ [eV]**	**ΔH [eV]**	**ΔS [eV]**	**$\Delta E_{D3,corr}$ [eV]**	**ΔG^{H+}_{corr} [eV]**	**$\Delta G_{TZ,rel}$ [eV]**	**Notes**
	**Mn_B-**	**Mn_A-**								
Mn4z44	O*	OH	22.40	22.16	6.21	0.0101	-2.23	22.2181	3.14	**OO1**
Mn44y4	O*	OH	22.01	21.77	6.22	0.0101	-2.23	22.2181	2.76	**OO2**
Mn44y4	$O^{0.5}$	OH	23.31	23.12	6.19	0.0100	-2.19	22.2181	4.10	**TS1**
Mnz4y4	OOH		22.10	22.00	6.26	0.0102	-2.17	22.2181	2.99	**3b**
Mnx444	OH	O*	22.37	22.11	6.20	0.0100	-2.23	22.2181	3.12	**OO3**
Mn44y4	OH	O*	22.08	21,83	6.23	0.0100	-2.23	22.2181	2.85	**OO4**
Mn4zy4		OOH	21.93	21.83	6.26	0.0102	-2.18	22.2181	2.83	**3a**
Mnzzy4	-OO*H-		22.42	22.47	6.18	0.0102	-2.20	22.2181	3.41	**TS3**

VI. Cartesian coordinates of selected intermediates. transition states

44

Intermediate 1

Mn	-0.552281	1.412095	0.711433
Mn	-2.629225	0.003653	-0.524445
Mn	-0.109996	0.092820	-1.908274
Mn	-0.533718	-1.507237	0.552272
O	-1.736558	-0.076282	1.061308
O	-1.385345	1.288602	-1.027352
O	0.539303	-0.005236	0.002263
O	-1.380574	-1.197849	-1.179755
H	-3.830650	-4.238383	1.816387
H	-5.193739	-3.202502	1.221646
C	-3.237120	-2.537679	0.647727
C	-4.210649	-3.630002	0.986619
O	-2.015094	-2.736186	0.928948
O	-3.706561	-1.499678	0.075191
H	-4.315577	-4.272420	0.096610
O	-2.058694	2.579084	1.209260
O	-3.725714	1.431685	0.196214
O	1.004988	1.480728	-2.313738
V	1.788055	2.399554	-1.053481
O	2.306593	3.759763	-1.649513
O	0.572075	2.730773	0.156709
O	0.607271	-2.741835	-0.129865
V	1.843922	-2.259874	-1.272934
O	1.073069	-1.214508	-2.423304
O	2.387522	-3.537399	-2.014642
O	1.218047	-0.242719	4.369986
V	1.088087	-0.151235	2.805224
O	0.168715	1.272958	2.386941
O	0.185029	-1.534298	2.227406
O	2.729047	-0.077264	2.078678
O	5.095319	0.016420	0.813248
V	3.549841	0.003132	0.526285
O	3.200814	-1.416393	-0.447618

O	3.156245	1.483921	-0.341247
H	-3.849274	4.084743	2.099618
H	-4.493089	4.077297	0.425948
C	-4.264463	3.453409	1.305503
C	-3.271368	2.410129	0.881655
H	-5.196907	2.970296	1.627510
O	-1.085352	0.178520	-3.462824
O	-3.447963	0.117431	-2.354233
H	-0.836255	0.988741	-3.940820
H	-3.945582	0.940417	-2.500182
H	-2.632109	0.155228	-2.964027

43

Intermediate 2b

Mn	0.529185	-1.457444	0.609933
Mn	2.630984	-0.002497	-0.573741
Mn	0.095849	0.054342	-1.992355
Mn	0.540890	1.407511	0.686814
O	1.724228	-0.039699	1.074819
O	1.358213	-1.218225	-1.151569
O	-0.582621	-0.006067	0.181038
O	1.356068	1.261281	-1.078302
H	3.868599	4.108542	2.049925
H	5.222172	3.061076	1.458997
C	3.265683	2.449293	0.815202
C	4.253421	3.515014	1.211674
O	2.052584	2.630011	1.128142
O	3.729898	1.456669	0.164960
H	4.399967	4.178865	0.343624
O	2.013841	-2.710855	1.004233
O	3.715632	-1.495945	0.142575
O	-1.096112	-1.316947	-2.463023
V	-1.827310	-2.300866	-1.253144
O	-2.380445	-3.625482	-1.918890
O	-0.626437	-2.724098	-0.063354
O	-0.604773	2.723360	0.093313

V	-1.806181	2.383884	-1.121439
O	-1.078972	1.466054	-2.384033
O	-2.345466	3.750795	-1.708809
O	-1.254390	-0.120050	4.345976
V	-1.081718	-0.073978	2.778287
O	-0.172166	-1.494680	2.318161
O	-0.157249	1.358738	2.401163
O	-2.750286	-0.039896	2.079128
O	-5.095350	0.001891	0.785346
V	-3.541465	0.002069	0.510081
O	-3.180719	1.470322	-0.379041
O	-3.192132	-1.421149	-0.454082
H	3.865243	-4.082843	2.067089
H	4.245083	-4.335056	0.344000
C	4.210741	-3.585288	1.151631
C	3.238941	-2.508289	0.743265
H	5.216644	-3.167504	1.283932
O	1.514024	0.017308	-3.772598
O	3.541248	0.051845	-2.200657
H	2.414840	0.016456	-3.252207
H	4.026590	0.891322	-2.265298

43

Intermediate 2a

Mn	0.589930	-1.489423	0.535132
Mn	2.847260	-0.009554	-0.545729
Mn	0.097833	0.013130	-1.820422
Mn	0.619654	1.433729	0.602417
O	1.792860	-0.048227	1.005637
O	1.273105	-1.296735	-1.203018
O	-0.529777	-0.006558	-0.033125
O	1.324860	1.277740	-1.131701
H	3.759640	4.705560	0.627552
H	3.817860	4.072854	2.285078
C	3.223520	2.650496	0.790142
C	4.045744	3.833239	1.236785

O	1.979682	2.703891	1.099285
O	3.776218	1.724419	0.151798
H	5.115594	3.630394	1.107445
O	1.957563	-2.784094	0.965388
O	3.746050	-1.756754	0.047801
O	-1.126955	-1.296309	-2.374061
V	-1.877017	-2.301411	-1.185849
O	-2.443390	-3.607259	-1.877117
O	-0.653446	-2.744169	-0.035857
O	-0.594114	2.729431	0.061084
V	-1.803118	2.362938	-1.128791
O	-1.045432	1.406085	-2.353590
O	-2.336211	3.710109	-1.764578
O	-1.134542	-0.093838	4.380534
V	-1.017511	-0.056880	2.801882
O	-0.146178	-1.447594	2.258211
O	-0.120528	1.339610	2.321282
O	-2.683878	-0.003485	2.084899
O	-5.091240	0.053757	0.894945
V	-3.547788	0.027312	0.562890
O	-3.181950	1.470443	-0.368098
O	-3.236633	-1.421990	-0.375521
H	4.161551	-3.805075	2.210220
H	3.541310	-4.814743	0.887537
C	4.048405	-3.866818	1.115892
C	3.198626	-2.707561	0.661160
H	5.037620	-3.828994	0.644251
O	1.173331	0.087001	-3.529894
O	3.557196	0.020727	-2.336779
H	1.055840	0.971077	-3.918362
H	2.138091	0.051026	-3.205743

42

Intermediate 3a

Mn	0.575484	-1.497137	0.769574
Mn	2.585301	0.036227	-0.578147

Mn	0.062460	-0.113873	-1.904736
Mn	0.420859	1.472758	0.541953
O	1.638744	0.092415	1.070347
O	1.370827	-1.289287	-1.052438
O	-0.744066	0.182759	0.069633
O	1.283605	1.215254	-1.188411
H	3.762155	4.315012	1.809384
H	5.130840	3.282420	1.234025
C	3.173094	2.597170	0.640169
C	4.146941	3.701885	0.984446
O	1.960140	2.774905	0.933704
O	3.663941	1.581351	0.048996
H	4.266207	4.344071	0.096130
O	2.418319	-2.616980	1.246330
O	3.861962	-1.248465	0.184773
O	-1.040543	-1.583536	-2.351014
V	-1.767830	-2.490283	-1.076076
O	-2.343728	-3.853618	-1.663489
O	-0.607950	-2.846225	0.142982
O	-0.692721	2.806260	-0.187193
V	-1.850415	2.220004	-1.341048
O	-1.110521	1.224407	-2.548484
O	-2.449155	3.492993	-2.079214
O	-1.366565	0.263070	4.337254
V	-1.157836	0.145166	2.766258
O	-0.269650	-1.298744	2.470366
O	-0.282112	1.567290	2.266086
O	-2.833108	0.084408	2.048951
O	-5.101725	-0.080459	0.687056
V	-3.536377	-0.026916	0.446797
O	-3.272309	1.391804	-0.553081
O	-3.138170	-1.529207	-0.369765
H	4.442158	-3.946569	1.957755
H	5.229613	-3.534243	0.394456
C	4.754166	-3.125796	1.300160
C	3.567001	-2.276395	0.893211

H	5.502412	-2.496532	1.805394
O	3.458359	-0.024894	-2.234810
O	2.534688	-0.309186	-3.285227
H	2.397259	-1.268543	-3.174380

42

Intermediate 3b

Mn	-0.630907	1.536953	0.725979
Mn	-2.711719	-0.037049	-0.580077
Mn	-0.157371	-0.026190	-1.850350
Mn	-0.506940	-1.399368	0.658171
O	-1.613462	-0.002928	1.212451
O	-1.411588	1.213818	-1.097538
O	0.636709	-0.156560	-0.072800
O	-1.366203	-1.295013	-1.077391
H	-3.739804	-4.303783	1.963671
H	-5.116519	-3.172931	1.668025
C	-3.246369	-2.526365	0.829349
C	-4.205442	-3.619415	1.244124
O	-2.033686	-2.658483	1.157248
O	-3.750699	-1.565255	0.162751
H	-4.501566	-4.182084	0.343991
O	-2.496293	2.704499	1.051758
O	-3.960025	1.350613	-0.003220
O	0.994954	1.361244	-2.368583
V	1.719899	2.389001	-1.183197
O	2.273797	3.696623	-1.903834
O	0.564116	2.845204	-0.002590
O	0.618853	-2.804680	0.019406
V	1.794648	-2.291505	-1.136818
O	1.051853	-1.383859	-2.404705
O	2.405975	-3.602953	-1.797228
O	1.376942	0.001122	4.408341
V	1.174920	0.048385	2.829767
O	0.297796	1.458722	2.417020
O	0.329112	-1.384888	2.336406

O	2.827183	0.081986	2.065331
O	5.120250	0.125509	0.694393
V	3.550926	0.072042	0.470796
O	3.224273	-1.409892	-0.394695
O	3.126816	1.518284	-0.424731
H	-4.468912	4.193345	1.561907
H	-5.208231	3.714908	-0.006307
C	-4.795988	3.346393	0.946265
C	-3.641995	2.409327	0.655779
H	-5.599366	2.793471	1.456354
O	-1.097922	-0.048963	-3.485739
O	-2.481935	-0.328893	-3.277877
H	-2.477015	-1.295539	-3.149816

42

Intermediate 4b-I

Mn	0.646494	1.178519	-1.179735
Mn	2.693273	0.024442	0.501067
Mn	0.152812	0.555575	1.746969
Mn	0.447513	-1.579820	-0.242432
O	1.604449	-0.452752	-1.199375
O	1.471824	1.447196	0.660609
O	-0.706079	-0.206488	-0.086786
O	1.341880	-0.937495	1.392199
H	3.633371	-4.855382	-0.490783
H	5.031159	-3.720211	-0.615433
C	3.186361	-2.788306	-0.017070
C	4.122952	-3.977780	-0.051036
O	1.972561	-2.988012	-0.286950
O	3.718643	-1.674400	0.302507
H	4.432251	-4.210461	0.980808
O	2.548407	2.151788	-1.883999
O	3.992874	1.127452	-0.485057
O	-0.947234	2.119173	1.826374
V	-1.652943	2.684473	0.365936
O	-2.198238	4.169468	0.584786

O	-0.493700	2.694094	-0.900321
O	-0.688677	-2.690154	0.833492
V	-1.818491	-1.816182	1.802008
O	-1.060792	-0.563329	2.712551
O	-2.443652	-2.851538	2.841153
O	-1.425550	-1.348394	-4.184211
V	-1.182551	-0.841228	-2.692979
O	-0.284898	0.616738	-2.786957
O	-0.344047	-2.121084	-1.858527
O	-2.855083	-0.563292	-2.003466
O	-5.082031	-0.007125	-0.673224
V	-3.508149	-0.039976	-0.464816
O	-3.243203	-1.178692	0.838932
O	-3.059224	1.609601	-0.065947
H	4.548392	3.374806	-2.817751
H	5.364593	3.278337	-1.216531
C	4.870770	2.701266	-2.013916
C	3.692120	1.945519	-1.429804
H	5.610307	1.982608	-2.400005
O	1.340003	0.990425	3.817284
O	2.188235	1.986538	3.744422
H	2.601728	2.057458	4.634584

42

Intermediate 4a-I

Mn	-0.496882	1.501583	0.789336
Mn	-2.650040	-0.019924	-0.419231
Mn	-0.153666	0.069018	-1.849305
Mn	-0.382718	-1.410385	0.652506
O	-1.464525	-0.030834	1.309708
O	-1.411891	1.265012	-1.024998
O	0.779273	-0.164122	0.054211
O	-1.346732	-1.229881	-1.055821
H	-3.548187	-4.458725	1.884807
H	-4.777541	-3.176950	2.164770
C	-3.137775	-2.550689	0.938302

C	-4.090181	-3.621669	1.427954
O	-1.909055	-2.704501	1.173927
O	-3.674922	-1.569328	0.329824
H	-4.701545	-3.979046	0.585458
O	-2.345291	2.679211	1.269136
O	-3.878888	1.354682	0.276476
O	0.949923	1.515360	-2.425706
V	1.735096	2.457558	-1.220059
O	2.281199	3.804931	-1.880050
O	0.645923	2.851809	0.047006
O	0.683991	-2.807330	-0.128006
V	1.768821	-2.276048	-1.358164
O	0.977113	-1.304341	-2.545061
O	2.318817	-3.578354	-2.095363
O	1.720174	-0.143012	4.272712
V	1.398717	-0.064406	2.713955
O	0.522236	1.382446	2.440692
O	0.495488	-1.498778	2.312058
O	3.032902	-0.041900	1.887224
O	5.188116	0.064694	0.342091
V	3.604496	0.021234	0.234484
O	3.248845	-1.427069	-0.684755
O	3.151951	1.516224	-0.567838
H	-4.268146	4.187442	1.895717
H	-5.183115	3.652118	0.440130
C	-4.646710	3.319058	1.342214
C	-3.514894	2.392569	0.940470
H	-5.367954	2.764434	1.962523
O	-4.058094	-0.173932	-2.386641
O	-3.335590	-0.103197	-3.476489
H	-3.965196	-0.176263	-4.229391

41

Intermediate 4b-II

Mn	-0.550004	1.451525	0.813313
Mn	-2.670412	0.001680	-0.591108

Mn	-0.110897	0.052593	-1.757121
Mn	-0.535140	-1.511848	0.718878
O	-1.587603	-0.050584	1.264830
O	-1.345866	1.281347	-0.995650
O	0.800038	0.009645	-0.188844
O	-1.332958	-1.235586	-1.073249
H	-4.365587	-4.330889	1.472246
H	-5.454873	-2.903149	1.560027
C	-3.562134	-2.499955	0.623652
C	-4.716383	-3.436962	0.940934
O	-2.417829	-2.787675	1.026340
O	-3.895228	-1.455893	-0.047443
H	-5.224598	-3.727189	0.008451
O	-2.446505	2.694519	1.177124
O	-3.908708	1.421954	0.014723
O	0.961690	1.486223	-2.396813
V	1.721386	2.372615	-1.115902
O	2.279668	3.721341	-1.765118
O	0.637111	2.831012	0.132840
O	0.662726	-2.833490	-0.052113
V	1.743055	-2.288170	-1.268594
O	0.977946	-1.325906	-2.490744
O	2.317535	-3.586838	-2.001186
O	1.589498	-0.136137	4.431368
V	1.269074	-0.088446	2.862254
O	0.382715	1.335114	2.515201
O	0.396308	-1.496193	2.424084
O	2.867174	-0.048193	1.970713
O	5.113763	-0.000454	0.570092
V	3.537550	0.002295	0.353906
O	3.221287	-1.454449	-0.569895
O	3.213841	1.510132	-0.481218
H	-4.383351	4.258972	1.603011
H	-5.071320	3.819768	0.000641
C	-4.711931	3.428204	0.965606
C	-3.577367	2.448477	0.713491

H	-5.556806	2.902454	1.436650
O	-1.370161	0.099674	-3.414423
O	-2.630161	0.086929	-3.084429

41

Intermediate 4a-II

Mn	-0.535256	1.543077	0.647750
Mn	-2.724586	0.027391	-0.587230
Mn	-0.026621	0.179011	-1.889929
Mn	-0.512377	-1.437512	0.461775
O	-1.692530	-0.037577	0.988116
O	-1.179409	1.448389	-1.131320
O	0.737904	-0.187397	0.072554
O	-1.289831	-1.151894	-1.255996
H	-3.973762	-3.921589	2.280279
H	-5.102777	-3.761142	0.891787
C	-3.215539	-2.703871	0.679218
C	-4.050093	-3.871685	1.182329
O	-1.965590	-2.798525	0.895651
O	-3.802773	-1.752144	0.109498
H	-3.645416	-4.815201	0.785449
O	-2.359347	2.741797	1.190320
O	-3.916192	1.467927	0.166843
O	1.246686	1.572367	-2.341959
V	1.960417	2.451995	-1.057568
O	2.630643	3.788331	-1.634095
O	0.795915	2.864801	0.125234
O	0.637969	-2.807163	-0.220281
V	1.819830	-2.245446	-1.347090
O	1.140306	-1.227609	-2.549735
O	2.409926	-3.533281	-2.086913
O	1.234172	-0.244930	4.320652
V	1.059095	-0.143071	2.735504
O	0.236441	1.329817	2.413568
O	0.172281	-1.542382	2.247222
O	2.780568	-0.161881	2.086614

O	5.117187	-0.119391	0.829103
V	3.556319	-0.088378	0.520872
O	3.263158	-1.474460	-0.510473
O	3.277101	1.434243	-0.296425
H	-4.266007	4.222085	1.924496
H	-5.143242	3.825407	0.405823
C	-4.651737	3.407177	1.298646
C	-3.535430	2.472091	0.861037
H	-5.414608	2.836406	1.850337
O	-2.964251	0.145772	-3.335144
O	-3.743662	0.065852	-2.291064

41

Intermediate 5

Mn	-0.463518	1.321857	0.971226
Mn	-2.688917	-0.029077	-0.277403
Mn	-0.336256	0.209641	-1.802527
Mn	-0.327766	-1.574098	0.648804
O	-1.439012	-0.233752	1.379400
O	-1.499453	1.349654	-0.758981
O	0.740133	-0.209721	-0.051525
O	-1.696004	-1.030742	-1.444167
H	-3.603732	-4.537441	1.862145
H	-5.040381	-3.530502	1.427718
C	-3.139556	-2.721883	0.773457
C	-4.054938	-3.888577	1.099618
O	-1.903721	-2.896510	0.951802
O	-3.702950	-1.678612	0.317570
H	-4.198674	-4.483628	0.182099
O	-2.332415	2.488558	1.740787
O	-3.897261	1.228666	0.708366
O	0.760784	1.736221	-2.319090
V	1.620172	2.537623	-1.074969
O	2.116592	3.964048	-1.619595
O	0.642972	2.781591	0.307332
O	0.678447	-2.856852	-0.388972

V	1.651489	-2.123039	-1.605845
O	0.785470	-1.093630	-2.685925
O	2.214872	-3.324165	-2.504238
O	2.098981	-0.264364	4.329873
V	1.600027	-0.239107	2.797811
O	0.592240	1.148081	2.579542
O	0.798348	-1.677157	2.466619
O	3.124007	-0.111902	1.751826
O	5.165069	0.066312	0.088953
V	3.570899	0.030808	0.073868
O	3.184278	-1.358189	-0.922742
O	3.106685	1.570270	-0.637606
H	-4.256321	3.830616	2.684387
H	-5.172107	3.538267	1.166160
C	-4.639719	3.059490	2.003790
C	-3.510502	2.199807	1.451708
H	-5.368960	2.422793	2.528331
O	-2.945042	0.652071	-4.117836
O	-3.975468	0.739403	-3.510731

39

Intermediate 6

Mn	-0.687150	1.468507	0.452743
Mn	-2.696248	-0.025618	-0.987648
Mn	-0.086663	-0.064554	-2.046634
Mn	-0.616488	-1.444987	0.608109
O	-1.787476	0.008813	0.889705
O	-1.370908	1.241355	-1.431418
O	0.621066	-0.226040	-0.076413
O	-1.552044	-1.206130	-1.789904
H	-4.125378	-4.197627	1.569220
H	-5.436524	-3.105399	0.978502
C	-3.445900	-2.512024	0.382502
C	-4.472283	-3.574253	0.735009
O	-2.269764	-2.683566	0.803589
O	-3.866154	-1.550016	-0.331939

H	-4.629552	-4.216908	-0.147060
O	-2.610850	2.756883	0.682151
O	-4.010318	1.381006	-0.435876
O	1.181842	1.336037	-2.535708
V	1.798679	2.329603	-1.288403
O	2.453803	3.641666	-1.941824
O	0.569440	2.805515	-0.198159
O	0.512098	-2.886805	-0.009468
V	1.726787	-2.354546	-1.109169
O	1.113258	-1.513707	-2.484937
O	2.408785	-3.685965	-1.682327
O	1.101607	0.389217	4.457958
V	0.911740	0.178691	2.871308
O	0.040532	1.531902	2.243327
O	0.117183	-1.277696	2.606346
O	2.612160	0.101115	2.142729
O	4.943938	0.022383	0.905969
V	3.382297	0.005598	0.582834
O	3.136725	-1.499849	-0.278482
O	3.121479	1.441912	-0.396606
H	-4.616797	4.249830	1.046747
H	-5.488932	3.534505	-0.359128
C	-4.931486	3.315336	0.564296
C	-3.734182	2.427923	0.251780
H	-5.615783	2.770793	1.235126

42

Intermediate OO1

Mn	0.567615	-1.414489	0.624027
Mn	2.643523	-0.000007	-0.687566
Mn	0.099814	-0.000050	-2.071490
Mn	0.567620	1.414521	0.623967
O	1.742100	0.000023	1.046970
O	1.332887	-1.244173	-1.144439
O	-0.575074	0.000008	0.196052
O	1.332896	1.244140	-1.144473

H	4.015544	3.848382	2.212043
H	5.298763	3.133178	1.157833
C	3.289724	2.458328	0.741336
C	4.270315	3.512700	1.197282
O	2.069722	2.661801	1.029541
O	3.753028	1.475343	0.091157
H	4.181138	4.380905	0.524145
O	2.069722	-2.661762	1.029625
O	3.753023	-1.475340	0.091188
O	-1.140467	-1.401292	-2.416915
V	-1.822403	-2.342604	-1.159456
O	-2.394486	-3.694320	-1.767966
O	-0.605750	-2.728737	0.015734
O	-0.605751	2.728736	0.015593
V	-1.822370	2.342541	-1.159609
O	-1.140400	1.401192	-2.417036
O	-2.394457	3.694226	-1.768179
O	-1.175812	0.000098	4.369360
V	-1.018829	0.000068	2.793112
O	-0.121830	-1.428135	2.368678
O	-0.121830	1.428248	2.368621
O	-2.712603	0.000076	2.128285
O	-5.074852	0.000054	0.869914
V	-3.520635	0.000025	0.570725
O	-3.186525	1.447224	-0.357425
O	-3.186556	-1.447271	-0.357278
H	4.015257	-3.848908	2.211751
H	4.181529	-4.380635	0.523653
C	4.270311	-3.512728	1.197230
C	3.289718	-2.458318	0.741378
H	5.298711	-3.133020	1.158278
O	1.278146	-0.000098	-3.783253
O	3.459208	-0.000024	-2.257304
H	2.727079	-0.000033	-2.955862

42

Intermediate OO2

Mn	-0.634384	1.549813	0.662806
Mn	-2.635277	-0.029925	-0.709299
Mn	-0.030974	0.009169	-1.895345
Mn	-0.517829	-1.434690	0.544823
O	-1.696318	-0.015028	0.986061
O	-1.344564	1.231106	-1.199338
O	0.658239	-0.165220	-0.106429
O	-1.312220	-1.271231	-1.198364
H	-3.966229	-3.841094	2.234650
H	-5.237947	-3.209989	1.116313
C	-3.233815	-2.502005	0.722725
C	-4.201723	-3.560160	1.198478
O	-2.012262	-2.683998	1.010275
O	-3.711101	-1.534979	0.058367
H	-4.071293	-4.457014	0.571534
O	-2.440701	2.702428	1.003860
O	-3.884834	1.303938	-0.015168
O	1.137654	1.405243	-2.303044
V	1.818438	2.413037	-1.074113
O	2.403516	3.726777	-1.749363
O	0.595085	2.852527	0.049970
O	0.634742	-2.803692	-0.057823
V	1.843312	-2.269901	-1.176530
O	1.107451	-1.346437	-2.462480
O	2.462862	-3.569365	-1.837750
O	1.158935	-0.088965	4.409672
V	1.038617	-0.019046	2.826848
O	0.180589	1.397814	2.386272
O	0.194468	-1.427427	2.267729
O	2.718932	-0.009098	2.131032
O	5.084438	0.047625	0.896691
V	3.533167	0.030573	0.583019
O	3.236791	-1.403804	-0.385173
O	3.179915	1.522383	-0.267475

H	-4.458552	4.055339	1.674539
H	-5.293789	3.549996	0.162264
C	-4.778145	3.198275	1.069267
C	-3.591795	2.348383	0.667548
H	-5.494262	2.581371	1.633253

42

Transition State TS2

Mn	0.610168	-1.513496	0.788423
Mn	2.647726	0.024478	-0.596651
Mn	0.153387	-0.054405	-1.877947
Mn	0.475283	1.447646	0.576520
O	1.649387	0.066379	1.126517
O	1.358224	-1.280799	-1.002530
O	-0.661716	0.168595	-0.088750
O	1.315199	1.275576	-1.117316
H	3.720623	4.492279	1.509273
H	4.906553	3.183145	1.834190
C	3.230007	2.560242	0.669499
C	4.227074	3.617651	1.083626
O	2.026519	2.719133	1.009573
O	3.711238	1.574289	0.014715
H	4.837542	3.908411	0.216243
O	2.520708	-2.629444	1.154813
O	3.930051	-1.293912	0.003526
O	-1.006934	-1.459514	-2.315967
V	-1.739278	-2.431562	-1.087082
O	-2.289039	-3.768817	-1.751557
O	-0.598429	-2.836794	0.128506
O	-0.652279	2.804653	-0.106822
V	-1.807195	2.220022	-1.260100
O	-1.042763	1.269500	-2.492702
O	-2.418233	3.495888	-1.981003
O	-1.357646	0.193970	4.409795
V	-1.154049	0.083223	2.835763
O	-0.260025	-1.338569	2.483834

O	-0.303038	1.487835	2.284358
O	-2.797316	0.014818	2.058996
O	-5.110086	-0.085121	0.729271
V	-3.546043	-0.048637	0.475571
O	-3.237479	1.380932	-0.485010
O	-3.147831	-1.532500	-0.367075
H	4.540408	-4.057098	1.668045
H	5.217328	-3.632164	0.056576
C	4.830347	-3.231479	1.006718
C	3.647557	-2.332265	0.716506
H	5.638407	-2.641582	1.465062

42

Intermediate OO3

Mn	0.593481	-1.443808	0.517659
Mn	2.842165	-0.009949	-0.607195
Mn	0.120058	-0.005791	-1.948719
Mn	0.611809	1.429322	0.525819
O	1.801732	-0.015248	0.963625
O	1.278477	-1.305718	-1.212314
O	-0.526681	0.000247	-0.006518
O	1.312067	1.273761	-1.205885
H	3.878646	4.674814	0.517790
H	3.821845	4.101737	2.197203
C	3.252491	2.645013	0.719161
C	4.095683	3.814954	1.172017
O	2.007898	2.728303	0.992709
O	3.810310	1.701945	0.103762
H	5.163498	3.572156	1.108525
O	1.993133	-2.749076	0.973756
O	3.788944	-1.719201	0.072140
O	-1.183462	-1.350670	-2.366969
V	-1.883744	-2.314045	-1.142716
O	-2.472180	-3.642643	-1.787968
O	-0.647629	-2.734525	0.000028
O	-0.610721	2.734018	0.003709

V	-1.851194	2.330551	-1.140566
O	-1.156183	1.366807	-2.365842
O	-2.425093	3.667629	-1.781685
O	-1.049978	-0.003235	4.386992
V	-0.957326	-0.002908	2.800421
O	-0.100961	-1.406101	2.289240
O	-0.086824	1.394356	2.290907
O	-2.656501	0.007820	2.138351
O	-5.081660	0.028177	0.988773
V	-3.539286	0.016231	0.627942
O	-3.217464	1.457163	-0.313288
O	-3.241147	-1.430910	-0.311698
H	4.200171	-3.678208	2.306367
H	3.609889	-4.753444	1.022738
C	4.099187	-3.788106	1.214428
C	3.235670	-2.659515	0.700435
H	5.094448	-3.758002	0.754338

42

Intermediate OO4

Mn	0.626211	-1.487875	0.773353
Mn	2.617296	0.005409	-0.631624
Mn	0.070258	-0.143404	-1.945116
Mn	0.494556	1.490918	0.436978
O	1.701698	0.103115	1.006489
O	1.365102	-1.302570	-1.060820
O	-0.646620	0.177666	-0.119728
O	1.351199	1.190080	-1.292395
H	3.741833	4.606165	1.079632
H	4.522979	3.350891	2.076841
C	3.223275	2.573294	0.582463
C	4.211603	3.614410	1.052409
O	2.001060	2.781014	0.830650
O	3.703972	1.554029	-0.003979
H	5.102715	3.618469	0.411434
O	2.478280	-2.610726	1.186065

O	3.889569	-1.293599	0.023297
O	-1.130925	-1.551381	-2.215970
V	-1.799676	-2.478067	-0.924343
O	-2.380305	-3.837165	-1.509265
O	-0.577963	-2.837125	0.232409
O	-0.631653	2.798479	-0.262825
V	-1.861329	2.158686	-1.321850
O	-1.148437	1.164896	-2.542154
O	-2.501409	3.403260	-2.066504
O	-1.150876	0.409149	4.389120
V	-1.042369	0.221857	2.815563
O	-0.180365	-1.223097	2.480304
O	-0.207013	1.595709	2.153011
O	-2.724845	0.137701	2.141910
O	-5.088736	-0.035974	0.907322
V	-3.536623	-0.016757	0.595077
O	-3.253637	1.354115	-0.455937
O	-3.163397	-1.550148	-0.164806
H	4.500061	-4.014119	1.755122
H	5.252955	-3.561971	0.182788
C	4.800749	-3.179167	1.110751
C	3.611508	-2.313033	0.761898
H	5.562167	-2.565102	1.615469

42

Transition State TS3

Mn	-0.358956	1.764853	0.363962
Mn	-2.652510	0.253905	-0.573303
Mn	-0.107760	-0.264419	-1.876800
Mn	-0.561146	-1.097590	0.924511
O	-1.510416	0.504644	1.159503
O	-1.262058	1.197030	-1.400896
O	0.727915	-0.150339	0.087942
O	-1.467185	-1.226414	-0.845079
H	-4.135509	-3.291165	2.745779
H	-5.405385	-2.175403	2.102834

C	-3.435649	-1.849852	1.289509
C	-4.503397	-2.754998	1.862125
O	-2.238549	-2.077957	1.616210
O	-3.840520	-0.946908	0.488681
H	-4.774469	-3.488699	1.084924
O	-2.056113	3.212698	0.450651
O	-3.712250	1.867649	-0.281437
O	1.159492	0.887882	-2.691881
V	2.015281	1.999518	-1.691736
O	2.721639	3.094759	-2.610185
O	0.936287	2.780194	-0.608103
O	0.351439	-2.732871	0.520453
V	1.515522	-2.613926	-0.749512
O	0.857106	-1.869825	-2.167842
O	1.926342	-4.100112	-1.143182
O	1.575066	0.757649	4.239059
V	1.304743	0.506827	2.691429
O	0.601084	1.926269	2.049363
O	0.242705	-0.882831	2.580171
O	2.939401	0.158209	1.959249
O	5.135158	-0.315623	0.542707
V	3.562004	-0.219182	0.365435
O	3.065957	-1.793011	-0.226061
O	3.297244	1.088603	-0.775544
H	-3.804005	5.029579	0.605571
H	-4.661756	4.303117	-0.798902
C	-4.257569	4.113324	0.207937
C	-3.240982	2.992612	0.129469
H	-5.100716	3.801084	0.842899
